# Retinopathy of prematurity: Metabolic risk factors

**DOI:** 10.7554/eLife.80550

**Published:** 2022-11-24

**Authors:** Zhongjie Fu, Anders K Nilsson, Ann Hellstrom, Lois EH Smith

**Affiliations:** 1 Department of Ophthalmology, Boston Children’s Hospital, Harvard Medical School Boston United States; 2 The Sahlgrenska Centre for Pediatric Ophthalmology Research, Department of Clinical Neuroscience, Institute of Neuroscience and Physiology, Sahlgrenska Academy, University of Gothenburg Gothenburg Sweden; Duke University School of Medicine United States; Icahn School of Medicine at Mount Sinai United States

**Keywords:** retinal metabolism, hyperglycemia, retinopathy of prematurity

## Abstract

At preterm birth, the retina is incompletely vascularized. Retinopathy of prematurity (ROP) is initiated by the postnatal suppression of physiological retinal vascular development that would normally occur in utero. As the neural retina slowly matures, increasing metabolic demand including in the peripheral avascular retina, leads to signals for compensatory but pathological neovascularization. Currently, only late neovascular ROP is treated. ROP could be prevented by promoting normal vascular growth. Early perinatal metabolic dysregulation is a strong but understudied risk factor for ROP and other long-term sequelae of preterm birth. We will discuss the metabolic and oxygen needs of retina, current treatments, and potential interventions to promote normal vessel growth including control of postnatal hyperglycemia, dyslipidemia and hyperoxia-induced retinal metabolic alterations. Early supplementation of missing nutrients and growth factors and control of supplemental oxygen promotes physiological retinal development. We will discuss the current knowledge gap in retinal metabolism after preterm birth.

## Introduction

Preterm birth is common worldwide. In addition to increased mortality, the short- and long-term complications arising from the arrest of normal development after premature birth include retinopathy of prematurity (ROP) as well as delayed physical growth, vascular abnormalities (including intraventricular hemorrhage), pulmonary disease, sepsis, poor neurocognitive development, and metabolic dysregulation with increased risk of diabetes in young adulthood (Ramel and Rao, 2020; Ramel et al., 2013). In humans, retinal vascular development begins in the second trimester of pregnancy and is complete around term (Roth, 1977). In infants who are born extremely preterm, before 28 weeks of gestation, the retina is incompletely vascularized at birth. The lower the gestational age (GA) age at birth, the less developed the neural retina and the larger the area of peripheral avascularity. Promoting physiological retinal neurovascular development after preterm birth would be of great benefit in preventing blinding neovascular ROP.

In Phase I ROP, which starts immediately after preterm birth, physiological retinal vascular growth is inhibited. The first metabolic disruption postnatally is excess oxygen; even room air can increase oxygen saturation (SpO_2_) above that in utero (Hutter et al., 2010; Lara-Cantón et al., 2022). More problematic is supplemental oxygen, often given to preterm infants to overcome poor lung function to reduce mortality. Hyperoxia inhibits normal retinal vascular development by suppressing oxygen-regulated vascular and neural growth factors. A balance must be found, with individualization of oxygen supplementation based on the GA, postnatal age and sex to optimize retinal and systemic outcomes (Oei et al., 2017; Oei and Vento, 2019; Vento et al., 2013). In addition to disruption of oxygen, there are metabolic fuel imbalances caused by relative starvation, hyperglycemia, and inadequate supplies of specific amino acids and lipids as well as hormonal imbalances, all of which inhibit normal vascular growth.

**Figure 1.**
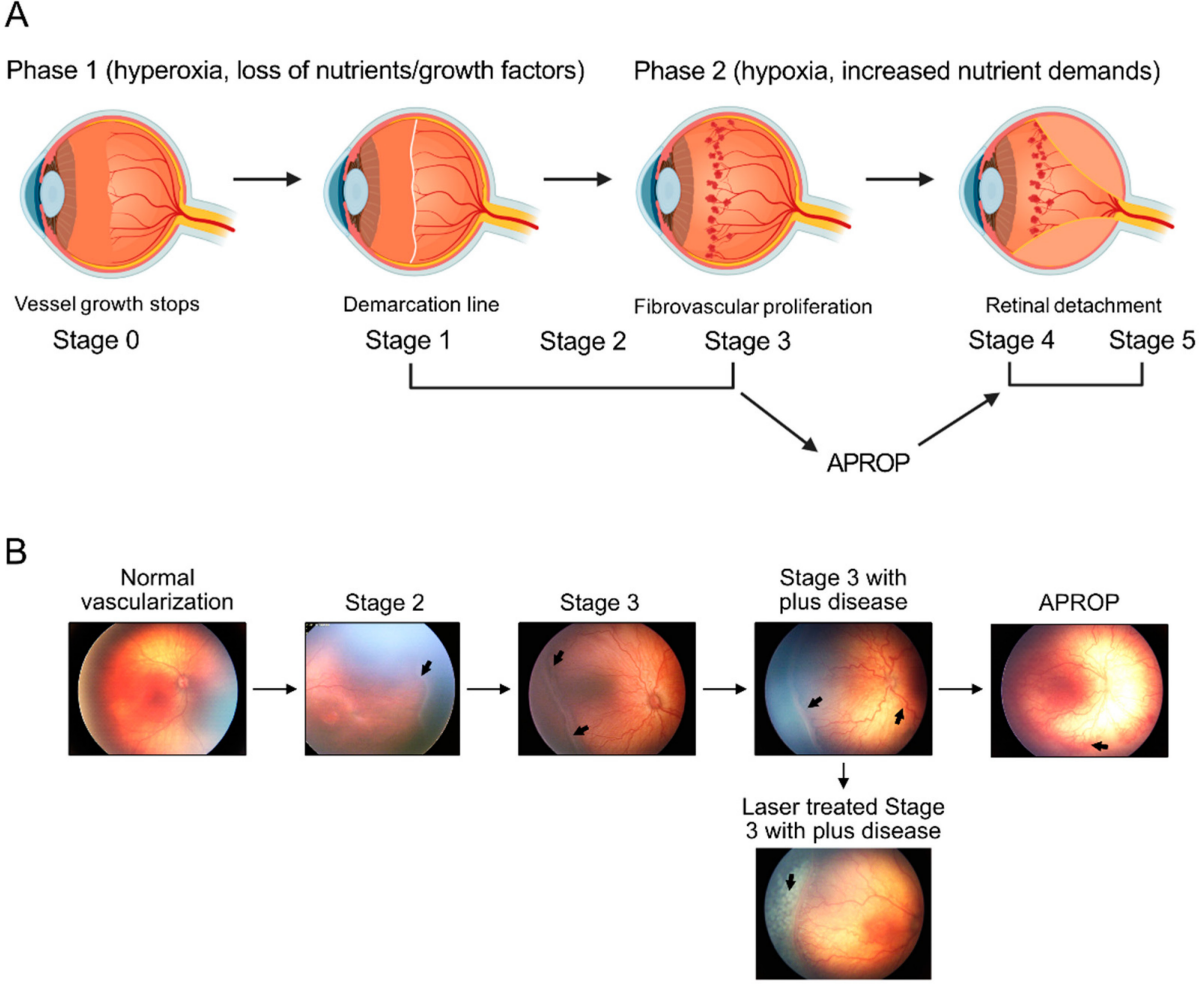
Schematics of ROP development (**A**) and illustration of human ROP development. In ROP, hyperglycemia and hyperoxia causes retinal vessel growth cessation (Phase 1). As the neural retina matures, increasing nutrient and oxygen demand triggers retinal neovessel growth (Phase 2). Human neovascular ROP (Phase 2) develops through the following stages: stage 2 with ridge (arrow), stage 3 with neovascularization and hemorrhage (arrows), stage 3 with plus disease (dilation and tortuosity of vessels) (arrow), Aggressive posterior ROP (APROP) with central changes (arrow) is particularly pathological. Laser treatment (arrow) of stage 3 ROP is illustrated. Figure was reproduced from Figure 1 from Tomita et al., 2021b.

In Phase II ROP, the avascular retina limits the delivery of both oxygen and nutrients causing hypoxia and fuel deficiency in the non-vascularized but slowly maturing retina. Retinal metabolism is also limited by a shortage of growth factors/nutrients normally provided by the mother during pregnancy which are missing after premature birth (Figure 1; Tomita et al., 2021b). Phase II ROP is driven by hypoxia and nutrient deficits that cause a massive release of vaso-formative factors triggering vision-threatening uncontrolled neovessel growth. Therefore, improving retinal vascularization during Phase I ROP will prevent the impairment in the vascular supply of oxygen and nutrients to meet the demand of growing neurons and prevents the progression to neovascularization of Phase II ROP.

### Retinal and choroidal vascular development

**Figure 2.**
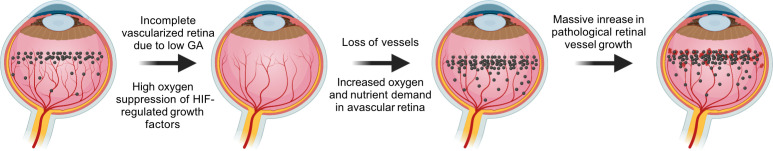
VEGF in the pathogenesis of ROP. During normal retinal vascular development, growth factor like VEGF (black dots) is found anterior to the developing vasculature driving the normal retinal vessel development forward. After preterm birth, in Phase I ROP, hyperoxia suppresses HIF-regulated growth factor (VEGF) production, causing vaso-obliteration and vessel growth cessation. As the retina matures with increasing metabolic demand, the non-perfused peripheral retina becomes hypoxic and nutrient deprived and overproduces growth factors (VEGF). Neovascularization occurs in response to high levels of growth factors. Images were created using BioRender, adapted and modified from ‘retina’, ‘dots’ by BioRender.com (2022).

To promote normal inner retinal vascularization, it is important to understand major imbalances in the normal metabolic driving forces. Vascularization of the maturing neural retina is normally stimulated by increased energy demands that create a wave of fuel and oxygen deficits moving from the optic nerve to the periphery as the retina matures that stimulates vaso-formative factors at the wave front. At the leading edge of the wave, the vaso-formative factors stimulate the physiological outgrowth of the vasculature which relieves the hypoxia and nutrient deficiency and locally suppresses the production of vaso-formative factors. In front of the wave, there is further maturation, further deficits of nutrients and oxygen, and further expression of vaso-formative factors (in particular vascular endothelial growth factor, VEGF) moving the vascularization process forward (Chan-Ling et al., 1995; Joyal et al., 2018; Joyal et al., 2016; Pierce et al., 1996; Shih et al., 2003). In the incompletely developed retina of a preterm child, oxygen supplementation can cause pruning of immature formed inner retinal vessels and suppression of new physiological vessel growth (Phase I ROP) (Figure 2).

**Figure 3.**
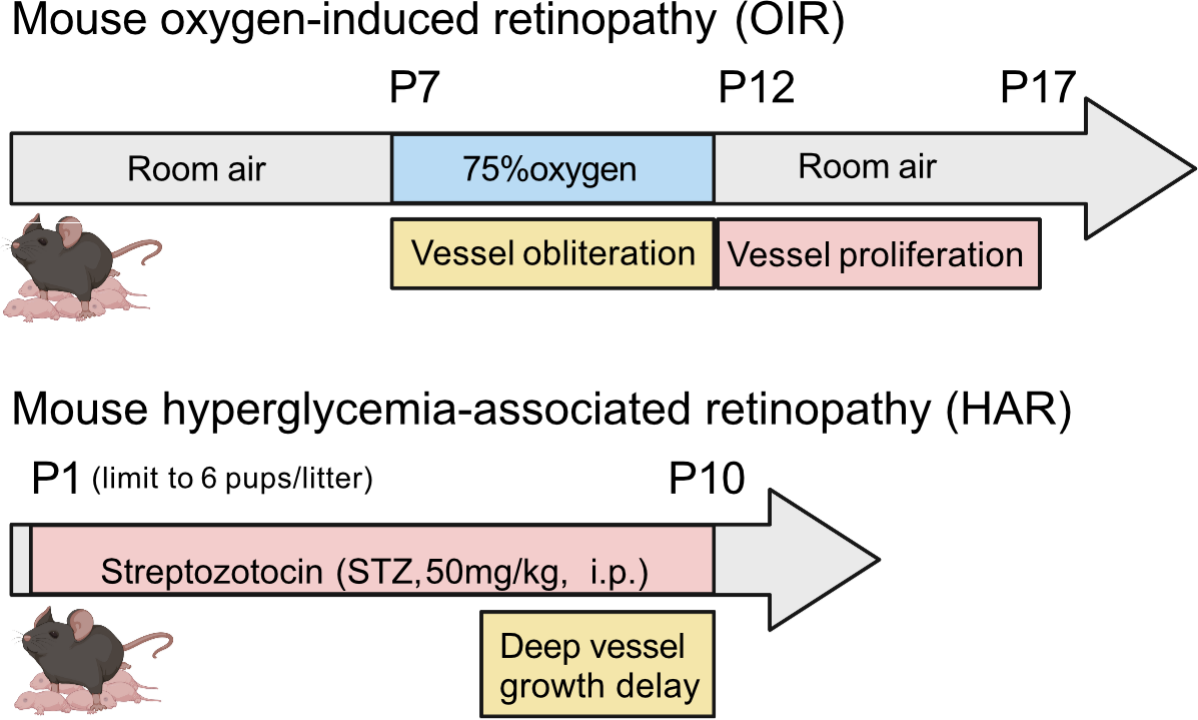
Schematics of mouse oxygen-induced retinopathy (OIR) and hyperglycemia-associated retinopathy (HAR). In OIR, mouse pups and the nursing dam are exposed to 75% oxygen from postnatal day (P) 7 for 5 days causing vessel loss and cessation of vessel growth and returned to room air where the avascular retina becomes hypoxic and causes neovascularization. There is also a metabolic model of suppression of retinal vessel growth as seen in Phase I ROP. In HAR, mouse pups are given low dose streptozotocin (STZ 50 mg/kg) daily from P1 to P9, causing hyperglycemia which suppresses normal vascular development examined at P10. Images were created using BioRender, adapted and modified from “mouse” by BioRender.com (2022).

There are two vascular systems in the retina. The inner retina is supplied by three layers of interconnected vessels that develop as described above. The outer retina, particularly the retinal pigment epithelium (RPE) and photoreceptors is supplied by a vascular plexus, the choriocapillaris, the precursors of which emerge at the fourth week of gestation. Most of the choroidal vasculature matures during the third and fourth months of gestation (Anand-Apte and Hollyfield, 2010). VEGFA is essential to maintain choroidal structure and function (Kim et al., 2020). In rodent oxygen-induced retinopathy (OIR) (Figure 3), the retardation of choroidal vascular development starts from postnatal day 7 during hyperoxia exposure and persists (Kim et al., 2018; Shao et al., 2011), suggesting that nutrient and oxygen supply from the choroidal vascular system to the RPE and photoreceptors is also affected with oxygen supplementation.

### Oxygen and fuel in the mature and immature retina

#### Oxygen gradients in the retina

**Figure 4.**
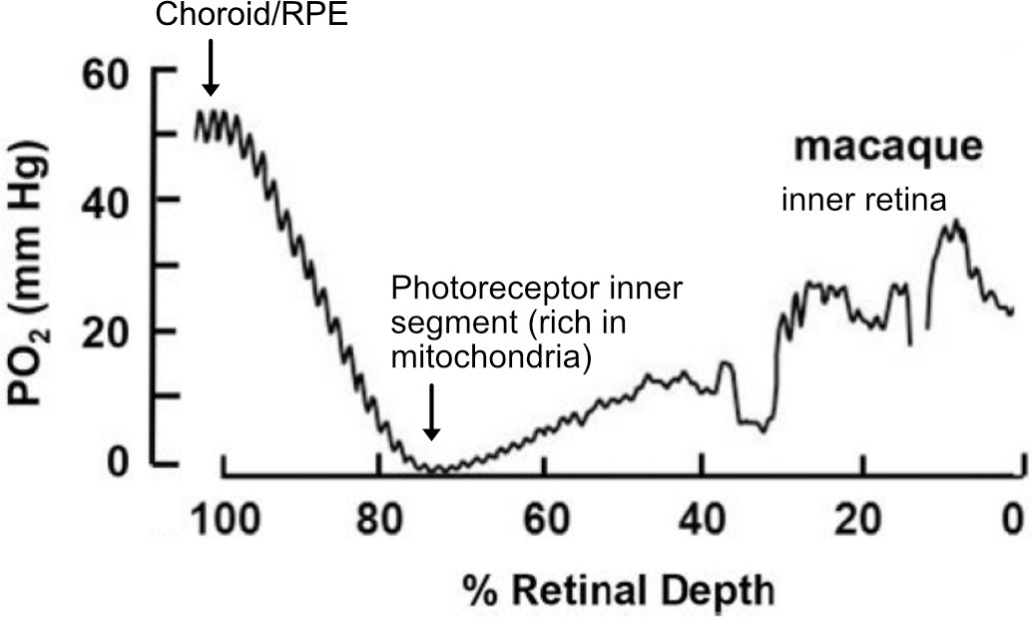
O2 profiles through the dark-adapted retina of rhesus monkey. A retinal depth of zero is the vitreoretinal border and 100% is the RPE-choroid border. The lowest oxygen tension is found in the layer containing photoreceptor inner segments with very high mitochondrial density. Rising oxygen tension across the inner retinal layers is due to three layers of inner retinal vasculature. Figure was reproduced from Figure 1 from Linsenmeier and Zhang, 2017. License number 5416050680408.

The normal mature vascularized retina is highly metabolically active and consumes oxygen avidly (Wangsa-Wirawan and Linsenmeier, 2003). Oxygen tension falls steeply from the choriocapillaris plexus to the photoreceptor inner segments, where SpO_2_ is close to zero, likely secondary to very high flux (Figure 4; Linsenmeier and Zhang, 2017). The SpO_2_ then increases gradually across the outer retina. In the inner retina, oxygen peaks are present close to inner retinal vessels (Cringle et al., 1991; Linsenmeier, 1986). Inner retinal oxygen tension is regulated through vaso-constriction of vessels to control blood flow (Riva et al., 1983). With high oxygen supplementation causing hyperoxia, the choroidal oxygen tension increases dramatically and a greater portion of the retina can then be supplied by the choroid (Cringle and Yu, 2018; Cringle et al., 1991; Linsenmeier and Yancey, 1989). Under conditions of hypoxia, the inner retina oxygen tension is regulated with increased retinal blood flow (Ahmed et al., 2001; Eperon et al., 1975; Palkovits et al., 2014; Wangsa-Wirawan and Linsenmeier, 2003). In the choroid however, hypoxia leads to a steep decrease in oxygen tension and causes a large decrease in photoreceptor oxygen consumption in dark-adapted retina but only mild changes are seen in light-adapted retina, which consumes less energy than in the dark (Joyal et al., 2018; Linsenmeier and Braun, 1992; Wangsa-Wirawan and Linsenmeier, 2003).

#### Hyperoxia, hypoxia, and ROP

Supplemental oxygen, besides increasing the risk of ROP, increases the level of reactive oxygen species (ROS), exceeding the capacity of the immature antioxidant defense system in preterm infants. Excess ROS damages DNA, RNA, lipids, proteins, membranes, and organelles. In premature infants with ROP vs. no ROP, there are lower levels of the antioxidants superoxide dismutase and glutathione in mitochondria (Lynch et al., 2016; Oziebło-Kupczyk et al., 2006) and higher total levels of pro-oxidants and malondialdehyde (Banjac et al., 2018). Modulation of redox homeostasis may help control cellular damage and should be considered to prevent ROP and other complications of preterm birth.

Low levels of oxygen are also damaging. Hypoxia induces nitric oxide synthases (NOSs) and releases NO (Jung et al., 2000), which is a strong competitor of oxygen for cytochrome c oxidase in the electron transport chain. Cells also decrease protein synthesis and Na-K-ATPase activity (a major ATP consumer) to reduce ATP demand by modulating AMP-activated protein kinase (AMPK) and mammalian target of rapamycin (mTOR) complex 1 (mTORC1) under hypoxic conditions (Gusarova et al., 2011). These hypoxia-induced adaptive pathways have been examined in mouse OIR retinas. Hypoxia (via induction of *Adora2a*) promotes endothelial cell glycolytic enzyme expression (Liu et al., 2017). Inhibition of endothelial NOS (eNOS) and inducible NOS (iNOS) inhibits retinal neovascularization (Brooks et al., 2001; Ninchoji et al., 2021; Zhang et al., 2009). iNOS expressed in the avascular retina increases retinal neuronal cell death (Sennlaub et al., 2002). pNaKtide, which inhibits Na-K-ATPase ROS amplification, reduces retinal neovascularization by decreasing HIF and VEGF levels. mTOR inhibitors (rapamycin and everolimus) reduce retinal neovascular tuft formation and rapamycin decreases the activation of cyclin D1 (an important regulator of cell cycle progression) in OIR retinas (Jiang et al., 2020; Yagasaki et al., 2014).

Since Phase I ROP involves oxygen-induced suppression of VEGF, which directs physiological vascularization, another approach to prevent Phase I ROP is to stabilize the upstream control of VEGF and other oxygen-regulated factors, including hypoxia-induced factor 1 (HIF1). HIF is degraded with hyperoxia, decreasing physiological VEGF production and suppressing physiological vessel growth. Stabilization of HIF1 with prolyl hydroxylase inhibition (systemic Roxadustat) in hyperoxia-induced Phase I ROP (OIR) suggests that serine and one-carbon metabolism may protect against vaso-obliteration (Singh et al., 2019). However, Phase II ROP can potentially be exacerbated with the stabilization of HIF1. In Phase II ROP, hypoxia in the avascular retina leads to stabilization of HIF1 protein and increased VEGF expression.

Hyperoxia and hypoxia through HIF alter multiple metabolic pathways. Hypoxia-induced HIF stabilization not only increases VEGF production to trigger new vessel growth to deliver more oxygen, but also modulates mitochondrial function to adapt to low oxygen status (Solaini et al., 2010; Wheaton and Chandel, 2011). In addition to mediating oxygen effects on the retina, HIF also affects energy metabolism through glycolysis and mitochondrial oxidative phosphorylation (OXPHOS) (Solaini et al., 2010; Wheaton and Chandel, 2011). HIF induces the expression of glucose transporters and glycolytic enzymes to enhance glycolysis and also activates pyruvate dehydrogenase kinase 1 and inactivates pyruvate dehydrogenase (Kierans and Taylor, 2021; Kim et al., 2006; Mobasheri et al., 2005), thereby preventing the conversion of pyruvate to acetyl-CoA for mitochondrial function.

Taken together, changes in oxygen alter multiple metabolic pathways and modulation of these pathways may help to prevent pathological retinal angiogenesis. Control of oxygen in Phase I ROP is essential to prevent suppression of retinal vessel growth and the progression to Phase II ROP (Oei et al., 2017; Oei and Vento, 2019; Vento et al., 2013).

#### Fuels used in retina

##### Glucose metabolism in the retina

###### Glucose as photoreceptor fuel

Glucose is the major fuel source for the retina, particularly photoreceptors, but it is primarily metabolized through aerobic glycolysis rather than OXPHOS despite the high density of mitochondria in photoreceptors (Cohen and Noell, 1960; Joyal et al., 2018). Photoreceptors lack direct contact with blood vessels, but adjacent RPE cells which are in contact with the high flow choroidal vasculature preferentially pass glucose to them (Kanow et al., 2017) while RPE itself can use fatty acids, amino acids, succinate and lactate both from the blood and from the retina as fuel (Hurley, 2021). In animal studies, when RPE is genetically manipulated to consume glucose, thereby decreasing glucose transport from RPE to photoreceptors, photoreceptors die (Zhao et al., 2011). Disruption of rod photoreceptor glycolysis with rod-specific knockdown of the rate-limiting glycolytic enzyme hexokinase 2 decreases photoreceptor function and increases photoreceptor mitochondrial mass, suggesting that OXPHOS is increased under the metabolic stress of decreased glycolysis (Petit et al., 2018). There is additional evidence that photoreceptors use glucose primarily for aerobic glycolysis to produce lactate (Joyal et al., 2018). Photoreceptors express high levels of glycolytic enzymes favoring lactate production, which is mainly transported to RPE and Müller glia as energy sources instead of being metabolized in photoreceptors (Country, 2017).

###### Hyperglycemia in ROP

Although glucose is the major fuel of photoreceptors, excess glucose is damaging. Hyperglycemia in the early postnatal period strongly influences the incidence and severity of ROP (Blanco et al., 2006; Chavez-Valdez et al., 2011; Mohsen et al., 2014). Preterm infants are born with an immature gastrointestinal tract that limits enteral nutrition, which in combination with low body fat reserves and high metabolic demands, may result in inadequate nutrition (starvation) (Webbe et al., 2022). Starvation triggers insulin resistance, hepatic gluconeogenesis, and hyperglycemia (Whitfield and Hendrikson, 2006). Postnatal hyperglycemia is found in ~80% of premature infants with birth weights of less than 750 grams and ~45% with birth weights of less than 1000 grams (Binder et al., 1989).

Animal models of metabolic influences on Phase I ROP have increased our understanding of the impact of hyperglycemia and dyslipidemia on postnatal retinal vascular development. In the hyperglycemia models of Phase I ROP in neonatal (although not preterm) rodents, as opposed to preterm infants, animals are metabolically developed and not starved and the response to hyperglycemia may not be the same. Streptozotocin (STZ)-induced postnatal hyperglycemia only partially mimics preterm hyperglycemia which results from both insulin deficiency and insulin resistance (Salis et al., 2017) and immature metabolic pathways (Whitfield and Hendrikson, 2006). In the eye in both neonatal rats and mice, hyperglycemia attenuates retinal vessel growth, increases inflammation and disrupts glucose metabolism (Fu et al., 2018a; Kermorvant-Duchemin et al., 2013). Hyperglycemia increases retinal apoptosis. There are fewer photoreceptors and other cells in the inner neuronal layer even after the hyperglycemia is resolved. In mice, neonatal hyperglycemia (Figure 3) suppresses the expression of genes involved in metabolism and there is delayed growth of all major retinal neurons (Fu et al., 2022). Decreased retinal neuronal activity (and cell number) persist into adulthood (Fu et al., 2018a). In mice, neonatal hyperglycemia also exacerbates hypoxia-induced retinal neovessel growth (Cakir et al., 2020). These findings correspond to clinical characteristics of ROP with hyperglycemia-associated retinal vascular growth delay in Phase I and increased disease severity in Phase II suggesting the utility of these Phase I ROP models despite the limitations noted above.

### Mitochondrial OXPHOS is necessary for eye function

Even though glucose is preferentially used as fuel by photoreceptors and glucose is primarily metabolized through aerobic glycolysis and not through mitochondrial OXPHOS, the very high density of mitochondria in the photoreceptor inner segment suggests that maintaining mitochondrial activity and OXPHOS is important to retinal function (Ball et al., 2022). There is genetic evidence of the importance of mitochondrial function in the eye. Leber hereditary optic neuropathy, affecting retinal ganglion cells is caused by a mutation in the *MT-ND5* gene (m.13345G>A), which affects mitochondrial complex I (Engvall et al., 2021). Other mitochondrial genetic disorders often have eye manifestations such as increased autofluorescence, macular dystrophy, yellow subretinal deposits, and abnormal electroretinogram (ERG) signals (Birtel et al., 2022; Daruich et al., 2014; Rath et al., 2008; Yu-Wai-Man and Newman, 2017), suggesting a disruption in RPE and photoreceptors.

Mitochondria in isolated mouse rod photoreceptors generate substantial nicotinamide adenine dinucleotide phosphate (NADPH) using glutamine in the absence of extracellular metabolic substrates which may support cell function under nutrient shortage (Adler et al., 2014). In zebrafish, overexpression of mitochondrial Ca^2+^ uniporter in cone photoreceptors enhances the TCA cycle and accelerates recovery kinetics of the cone response to light (Hutto et al., 2020), suggesting that improving photoreceptor mitochondrial energy production to prevent retinal neural dysfunction may be feasible. This concept is supported by the evidence that defects in ERG signals of mice lacking a subunit of mitochondrial complex I in vivo can be rescued by modulating the extracellular environment ex vivo when retinas are supplemented with proper nutrients (Gospe et al., 2019).

As glucose is mainly used for aerobic glycolysis in the retina, not mitochondrial OXPHOS, mitochondria also use alternative energy sources, particularly during stress conditions (Chinchore et al., 2017; Casson et al., 2016; Joyal et al., 2018; Joyal et al., 2016; Lindsay et al., 2014; Rajala et al., 2016; Reading, 1965; Reidel et al., 2011; Wang et al., 1997). Two major unanswered questions are (I) what fuels other than glucose are used in mitochondrial OXPHOS and (II) can these fuels or other molecules be provided to improve retinal metabolism in preterm infants?

#### Lipids

There is disrupted fatty acid oxidation in ROP infants (Yang et al., 2020). Lipid deficiency (including short-chain and long-chain fatty acids) in photoreceptors causes retinal degeneration and abnormal retinal vessel growth (Joyal et al., 2016). The saturated fatty acid palmitate (C16) has been shown to be a fuel source for OXPHOS in photoreceptors (Joyal et al., 2016). Other lipids have not yet been assessed.

Hypertriglyceridemia is correlated with an increased risk for ROP although the association becomes non-significant after adjustment for gestational age and birth weight (Sinclair et al., 2018). Moreover, blood proteins involved in lipid metabolism are associated with ROP (Danielsson et al., 2022). More than being a source for energy production, many lipids are bioactive and are involved in the regulation of diverse biological functions. Dietary essential omega-3 and omega-6 fatty acids are partly oxidized, but importantly, they also serve as substrates for the production of a large family of signaling molecules with metabolic and immune regulatory activities. Deficiency of omega-3 docosahexaenoic acid (DHA) and omega-6 arachidonic acid (ARA) in preterm infants causes developmental delay (Hadley et al., 2016; Smith and Rouse, 2017). The levels of circulating DHA, ARA, as well as the signaling sphingolipid sphingosine-1-phosphate (S1P) are inversely correlated with the risk of developing ROP (Hellström et al., 2021b; Löfqvist et al., 2018; Nilsson et al., 2021; Table 1). Interestingly, ARA vs. DHA better preserves retinal metabolism in mice with postnatal hyperglycemia (Fu et al., 2022).

**Table 1.** Summary of metabolic risk factors for ROP.

Risk factors	Comparison	Outcomes	References
DHA	no ROP, mild or moderate ROP (stage 1–2), or severe ROP (stage 3 and type 1).	High serum DHA correlated with less severe ROP, only in infants with sufficiently high ARA levels	Hellström et al., 2021b
	Enteral DHA vs. placebo	No difference in any stage of ROP, but DHA lowered the relative risk for severe ROP.	Bernabe-García et al., 2019
ARA	ROP vs. no ROP	Low serum ARA correlated with ROP development	Löfqvist et al., 2018
DHA +ARA	Enteral DHA +ARA vs. no supplementation	DHA:ARA at 1:2 ratio lowered severe ROP (stage 3 and/or type 1).	Hellström et al., 2021a
Metabolites	ROP vs. no ROP	Higher levels of glycolytic intermediates (pyruvate, lactate), lower levels of TCA metabolites (citrate, aconitate, succinyl carnitine), higher malonyl carnitine (C3DC), glycine in ROP	Yang et al., 2022; Yang et al., 2020
Insulin	No or mild ROP (1–2) vs. severe ROP (3-4)	Insulin exposure was a stronger predictor for severe ROP than hyperglycemia per se	Kaempf et al., 2011
	No or mild ROP vs severe ROP (needing treatment)	Blood glucose >150 mg/ml and insulin exposure associated with severe ROP	Lee et al., 2016
IGF-1	No ROP, ROP (1,2, 3-4)	Low plasma IGF-1 correlated with high glucose levels and increased ROP severity	Cakir et al., 2020
APN	No ROP stage vs. any ROP	Low serum APN correlated with ROP; serum APN positively correlated with serum DHA	Fu et al., 2015a
	Plasma glucose tertiles and retinal vascular coverage in preterm infants	Low serum APN correlated with high glucose levels and delayed retinal vascularization	Fu et al., 2018a

Peroxisomes may play a role in supplying appropriate lipids for optimal retinal mitochondrial metabolism. Although mitochondria are the primary organelle for fatty acid oxidation, peroxisomes uniquely oxidize very long-chain fatty acids which cannot be processed by mitochondria into shorter chains that can be used. Peroxisomal disorders are associated with retinal ganglion cell loss, and retinal degeneration (Chen et al., 2022; Das et al., 2021). In Zellweger syndrome with mutations in *PEX* genes involved in peroxisomal biogenesis, there is loss of retinal cells (photoreceptors, retinal ganglion cells) and diminished ERG signals. Gene defects in peroxisomal α- and β-oxidation may also cause attenuated light responses, decreased visual acuity, and blindness (Chen et al., 2022; Das et al., 2021). Although omega-3 long-chain polyunsaturated fatty acids are primarily obtained from dietary sources, there is some endogenous production from shorter-chain precursors. Peroxisomes contribute to the endogenous conversion of precursor omega-3 fatty acids to DHA (Chen et al., 2022; Das et al., 2021). Oral DHA ethyl ester supplementation improves visual function in patients with peroxisomal disorders (Noguer and Martinez, 2010). Moreover, peroxisomes are key in maintaining redox homeostasis (Cipolla and Lodhi, 2017; Fransen et al., 2012). Preserving retinal lipid homeostasis by modulating lipid metabolism and supplementing proper types of lipids would likely benefit retinal health. More work is necessary to determine the specific interventions needed.

#### Amino acids

There is currently very limited direct evidence that amino acids serve as mitochondrial fuel in the retina, although gene mutations in endogenous serine synthetic enzyme phosphoglycerate dehydrogenase (*PHGDH*) are associated with macular degeneration (Gantner et al., 2019; Scerri et al., 2017). Amino acid (arginine, glutamine) metabolism is altered in hyperglycemia- and dyslipidemia-associated retinal disorders in adults and in preterm infants (Paris et al., 2016; Rhee et al., 2018; Tomita et al., 2021a). However, there may be abnormal amino acid profiles in preterm infants associated with ROP, along with higher than normal steady-state plasma levels of glycolytic intermediates (pyruvate, lactate) and intermediates associated with fatty acid metabolism, and lower than normal plasma levels of TCA metabolites (citrate, aconitate, succinyl carnitine) (Yang et al., 2022). This study found perturbed metabolism of lipids, arginine, glycine, serine and threonine, alanine and aspartate and glutamate. As parenteral versus enteral nutrition during the neonatal period has a profound impact on the serum metabolome (Nilsson et al., 2022; Vanhaesebrouck et al., 2008), further validation of longitudinal nutritional management, serum metabolome and ROP risks is needed. In mice modeling hypoxia-induced severe ROP, retinal changes include increased metabolites associated with lipids, glycine and serine and threonine metabolism (Tomita et al., 2021a). However, it remains to be determined if these adaptations in fatty acid and amino acid metabolism during hypoxia are transient or whether they have a long-term impact.

### Recycling pathways in ROP

Retinal health depends on well-coordinated uptake, recycling and processing of nutrients and metabolites between cell organelles and through the ‘cell death’ and autophagy pathways. The metabolic interactions among retinal mitochondria, peroxisomes, lysosomes as well as endoplasmic reticulum need to be investigated. Disturbance of function in any of these organelles causes metabolic and cellular stress. ROP is associated with increased redox imbalance and disruption of the antioxidant system (Banjac et al., 2018; Boskabadi et al., 2021; Kumar et al., 2008; Lynch et al., 2016; Oziebło-Kupczyk et al., 2006; Spierer et al., 2005), ultimately leading to cell death.

The common types of cell demise including apoptosis, autophagy, necrosis and senescence have been reported in ROP retinas (Beauchamp et al., 2001; Binet et al., 2020; Crespo-Garcia et al., 2021; Oubaha et al., 2016; Pesce et al., 2021; Sennlaub et al., 2002; Sprott et al., 2019). Targeting retinal apoptosis and necrosis protects against hypoxia-induced neurovascular damage in mouse OIR (Beauchamp et al., 2001; Grant et al., 2020; Narayanan et al., 2014; Sennlaub et al., 2002). Emerging evidence has shown that targeting senescence and autophagy protects the retina in animal models of hypoxia-induced retinopathy.

#### Senescence and ROP

Recently, it has been shown that senescent cells accumulate in neovascular tufts in proliferative mouse OIR. Pharmacological inhibition of cellular senescence promotes neovessel regression and normal revascularization (Binet et al., 2020; Crespo-Garcia et al., 2021; Oubaha et al., 2016). Targeting endothelial cell senescence might be a way to ameliorate retinal proliferation in Phase II ROP without suppressing normal retinal vascularization as occurs with anti-VEGF treatment.

#### Autophagy, and other recycling pathways

Autophagy, a process in which lysosomes degrade and recycle cellular components, is key in preserving retinal homeostasis and sustaining metabolic function. Defects in autophagy also interrupt the RPE degradation of the photoreceptor outer segment and the recycling of proteins and lipids (Villarejo-Zori et al., 2021). Restoring photoreceptor autophagy dysregulated by accumulated circulating lipids enhances mitochondrial function and inhibits pathological retinal angiogenesis in mice (Heckel et al., 2022). Dysregulated retinal autophagic markers are reported in rat OIR and a potential association between autophagy and necroptosis (not apoptosis) is also observed (Pesce et al., 2021). However, pharmaceutical inhibition of autophagy does not restore neural retinal function compromised in OIR (Pesce et al., 2021). Loss of autophagy protein 5 in endothelial cells impairs mitochondrial function, decreases mitochondrial ROS and reduces retinal neovascular tuft formation in mouse OIR (Sprott et al., 2019). Further investigations of the role of autophagic responses in controlling retinal pathology are needed.

### Hormonal influence in ROP: insulin, IGF-1, adiponectin, FGF21

#### Insulin

Glucose IV infusion is commonly used in premature infants to provide calories. However, preterm infants may be unable to use excess glucose properly due to immature regulation systems including insufficient insulin secretion and insulin insensitivity (Salis et al., 2017). Exogenous insulin is sometimes given to control hyperglycemia, but its use is controversial as it is generally ineffective at controlling hyperglycemia and insulin use is associated with increased mortality (Beardsall et al., 2008) and ROP (Kaempf et al., 2011; Lee et al., 2016). In preterm infants, other metabolic regulators like insulin-like growth factor-1 (IGF-1), adiponectin (APN) and FGF21 may better targets to control hyperglycemia (Table 1).

#### Insulin-like growth factor-1 (IGF-1)

##### IGF-1 deficiency in ROP

Loss of hormones as well as essential nutrients normally provided in utero also delays retinal vascularization. IGF-1, mainly derived from the liver, regulates body and retina growth (Daughaday and Rotwein, 1989; Liegl et al., 2016). IGF-1 levels fall immediately after birth in preterm infants and remain low for many weeks. IGF-1 is required for normal vessel growth in mice (Hellstrom et al., 2001) and low IGF-1 levels suppress VEGF activation of endothelial cell proliferation (phase I ROP) (Hellstrom et al., 2001; Smith et al., 1999). Low systemic IGF-1 levels correlate with a high risk for neovascular ROP (Cakir et al., 2020; Hård et al., 2013; Hellgren et al., 2021; Hellström et al., 2003; Hellstrom et al., 2001; Jensen et al., 2017). Low IGF-1 levels also correlate with low weekly platelet counts and ROP progression (Cakir et al., 2018; Hellgren et al., 2021; Jensen et al., 2018). Experimental studies demonstrate that IGF-1 supplementation before high oxygen challenge decreases retinal vessel loss and subsequent hypoxia-induced neovascularization in mice OIR (Vanhaesebrouck et al., 2009). Low levels of plasma IGF1 correlate with high plasma glucose in extremely preterm infants (Cakir et al., 2020). Experimental investigation also shows that induction of postnatal hyperglycemia in mouse OIR exacerbates retinal neovascularization and attenuates normal retinal vascularization (Cakir et al., 2020). Meanwhile, liver-derived IGF1 is reduced and recombinant human IGF-1 treatment improves normal retinal vasculature (Cakir et al., 2020). This finding suggests that replacing IGF-1 is a feasible approach to treat and prevent ROP.

##### Prediction of ROP based on IGF-1 and growth

Because circulating IGF-1 levels correlate with body growth and postnatal weight gain, poor postnatal weight gain during the first weeks of life can be substituted for IGF-1 levels to predict the development of ROP. WINROP, the first ROP prediction algorithm and online monitoring tool, was based on sex, GA, and both weekly weight and IGF-1 levels of preterm infants (Löfqvist et al., 2009). Later, WINROP was found to function well using only weight gain, omitting IGF-1 blood sampling (Hellstrom et al., 2009). As even accurate weight gain may be difficult to measure routinely in preterm infants, recently, DIGIROP using GA at birth, sex, standardized birth weight and age at the first sign of ROP was developed to predict the risk for severe ROP and shows high predicative ability in a contemporary Swedish cohort without using either IGF-1 or weight gain (Pivodic et al., 2022).

### Adiponectin (APN) and Fibroblastic Growth Factor 21 (FGF21)

In mice with hyperglycemia-associated retinopathy (Figure 3), modeling Phase I ROP with suppression of physiological retinal vascularization, adiponectin (APN) is induced in response to insulin shortage; APN supplementation promotes physiological retinal vessel growth and improves long-term neural retinal function (Fu et al., 2018a). In addition, serum levels of liver-derived fibroblast growth factor 21 (FGF21), which is a metabolic regulator of APN production and secretion (Holland et al., 2013; Talukdar et al., 2016), normally increases immediately after birth (Sánchez-Infantes et al., 2015). However, FGF21 levels are very low in premature infants (below the sensitivity of most assays) (Guasti et al., 2014; Mericq et al., 2014). FGF21 treatment suppresses hypoxia-induced retinal neovessel growth through APN and protects neurons in diabetic retinopathy in mice (Fu et al., 2017; Fu et al., 2018b). In a phase 2 a clinical trial of a long-acting Fc-FGF21 fusion protein (efruxifermin), the hepatic fat fraction was reduced in adult patients with non-alcoholic steatohepatitis (ClinicalTrials.gov NCT03976401) (Harrison et al., 2021). AKR-001, an Fc-FGF21 analog, increases insulin sensitivity and reduces circulating lipids in adult diabetic patients (Kaufman et al., 2020). FGF21 treatment may also be a promising approach in preterm infants to promote retinal maturation by modulating insulin sensitivity and lipid metabolism.

### Retinal-cell-specific contribution to vessel growth

**Figure 5.**
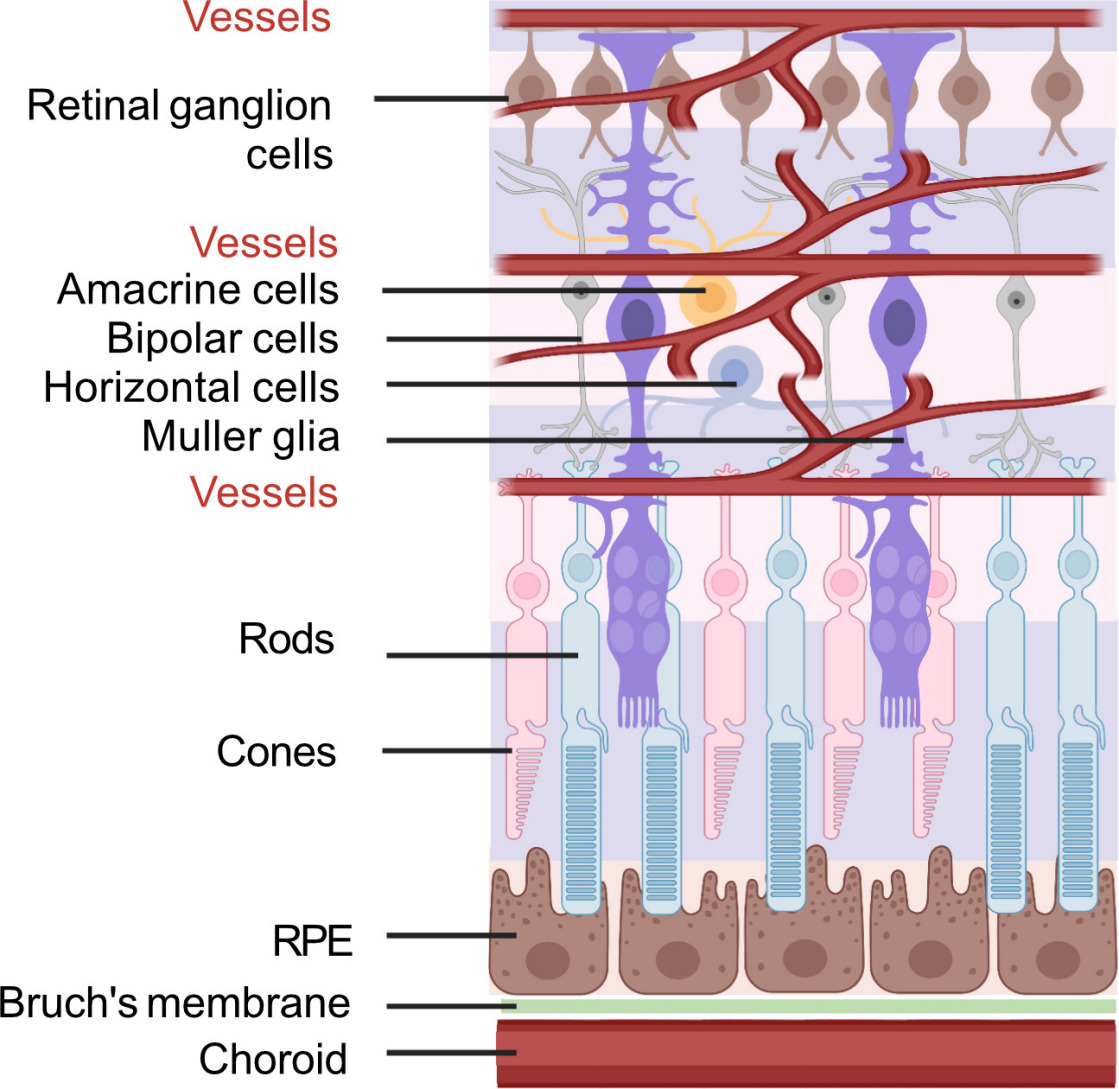
Schematic of retinal neuronal and vascular structure. RGC, retinal ganglion cells, RPE, retinal pigment epithelium. Images were created using BioRender, adapted and modified from ‘eye’, ‘retinal cell’, ‘generic branching vessel’ by BioRender.com (2022).

To better understand the retinal fuel demand in ROP, we also need to consider the retinal-cell-specific fuel preferences and metabolism as there are significant interactions among these cells. A schematic of retinal structure is shown in Figure 5.

#### Endothelial cells (EC)

Endothelial cell (EC) metabolism regulates proliferation and migration. EC glycolysis rather than OXPHOS generates ATP for vessel sprouting and loss of the rate-limiting enzyme in glycolysis 6-phosphofructo-2-kinase/fructose-2,6-biophosphatase 3 (PFKFB3) impairs tip cell formation (De Bock et al., 2013). Inhibition of EC glycolysis via targeting PFKFB3 or adenosine A2a receptor (ADORA2A) reduces retinal neovascularization in mice OIR (Liu et al., 2017; Schoors et al., 2014; Xu et al., 2014). Attenuation of the polyol pathway, which is induced under hyperglycemia, also decreases retinal neurovascular dysfunction in mouse OIR (Fu et al., 2015b; Fu et al., 2012). Increasing glycolysis by promoting glucose uptake during hyperoxia reduces retinal vessel loss and later neovascularization in rat OIR (Han et al., 2019). In addition to glycolysis, ECs rely on glutamine for vessel growth and blockade of glutamine use causes sprouting defects in physiological and pathological retinal angiogenesis (Huang et al., 2017). Loss of the endogenous serine synthetic enzyme PHGDH leads to EC death and impairs retinal angiogenesis (Vandekeere et al., 2018). Fatty acid oxidation and de novo lipogenesis also regulates EC proliferation and retinal vascular sprouting (Schoors et al., 2015; Wei et al., 2011). VEGF stimulation enhances the gene expression of fatty acid binding protein 4 (*Fabp4*) in ECs and loss of *Fabp4* in EC decreases proliferation (Elmasri et al., 2009).

#### Retinal neurons

Growing evidence has shown that retinal neurons control vessel growth (Fu et al., 2020). Photoreceptor dysfunction predicts vascular abnormalities in human ROP infants and rat OIR (Akula et al., 2007; Fulton et al., 2009). Photoreceptor glucose and fatty acid metabolism controls both physiological and pathological retinal angiogenesis (Fu et al., 2018a; Joyal et al., 2016). Photoreceptor c-FOS, a master inflammation regulator, modulates retinal neovascularization in mice (Sun et al., 2017). Moreover, retinal ganglion cells (RGCs) also control retinal vessel growth through G protein-coupled receptor-91 (GPR91) (Sapieha et al., 2008) and neuronal guidance cue semaphorin 3 A (SEMA3A) (Joyal et al., 2011), and capillary degeneration in mice induced with ischemia-reperfusion injury (Ueda et al., 2010; Zheng et al., 2007). Loss of retinal neuronal/glial suppressor of cytokine signaling 3 (SOCS3), which inhibits inflammation and VEGF signaling, exacerbates retinal angiogenesis in mouse OIR (Sun et al., 2015). Müller glia-derived VEGF is one of the primary driving forces of retinal neovascularization and leakage (Becker et al., 2018; Le, 2017; Wang et al., 2010). Stimulation of the G-protein-coupled receptor 81 (GPR81) in Müller glia with lactate induces angiogenic factor production and governs retinal vascularization (Madaan et al., 2019). Müller glial NETRIN-4, an axonal guidance molecule, increases VEGF release under hypoxic condition and NETRIN-4 stimulates proliferation in bovine retinal endothelial cells (Lange et al., 2012).

#### Microglia

Microglia are unique immune cells in the central nervous system necessary for homeostasis of the local microenvironment. Microglia control retinal vascular stability in normal and disease conditions (Arnold and Betsholtz, 2013; Davies et al., 2006). In mouse OIR, microglia are the predominant myeloid cells in neovascular tufts (Boeck et al., 2020). A study of the association between microglial status and retinal vessel growth in mouse OIR found that an increased number of microglia (mostly the activated amoeboid phenotype) in the superficial vascular layer correlates with increased superficial retinal vessels in OIR. However, an increase in microglia (mostly the quiescent ramified phenotype) in the deep vascular layer does not correlate with deep retinal vessels. Loss of microglia before and immediately after hyperoxia leads to more subsequent retinal neovascularization (Liu et al., 2022). These findings suggest a role for microglia in modulating retinal neovascularization.

In summary, in ROP eyes, there are shifts in glucose, lipid and amino acid metabolism, as well as disruptions in redox balance associated with cell death. Further studies regarding the long-term impact of metabolic imbalance on the neurovascular retina are needed. Metabolic crosstalk between different retinal cell types should be examined. RPE and Müller glia metabolically support photoreceptors. RPE and Müller glia have fuel preference that spare glucose for photoreceptors. Metabolic and molecular interaction among retinal organelles also need to be further examined to better understand their involvement in controlling retinal cell homeostasis.

### Gut microbiota and retinal development

At birth, the neonatal gastrointestinal tract is a sterile environment but quickly becomes colonized by microbes, that is, fungi, bacteria, archaea, protozoa, and viruses. The mother exposes the fetus to IgG and microbial antigens prenatally, and supplies the child with antibodies and metabolites through breast milk postnatally to protect against invasive pathogens and promote a beneficial microbiome (Brodin, 2022). Factors that influence the colonization process include GA (Fouhy et al., 2019), cesarian delivery (Fouhy et al., 2019), feeding type (mother’s own milk/donor milk/formula/parenteral nutrition) (Dahlgren et al., 2019; Kumbhare et al., 2022; Parra-Llorca et al., 2018; Piñeiro-Ramos et al., 2021), and exposure to antibiotics (Arboleya et al., 2015; Gibson et al., 2016). It is now widely accepted that the gut microbiome plays crucial roles in maintaining health homeostasis, and if disturbed (dysbiosis), can contribute to the pathogenesis of several diseases, including retinal disease (Rinninella et al., 2018), which has led to the concept of a ‘gut-retina axis’. Recent evidence also suggests that an imbalance in the neonatal gut microbiome can affect the clinical course of ROP.

In a pilot study, Skondra et al. found that infants with type 1 ROP compared with controls with no ROP had enrichment of stool *Enterobacteriaceae* at 28 weeks’ PMA (Skondra et al., 2020). The change in the gut microbiome was accompanied by alterations in microbial metabolic pathways, including amino acid biosynthesis, which was suggested to be related to infant IGF-1 expression. Interestingly, using a mouse model to investigate the effect of the gut microbiome on retinal gene expression, the signaling pathways of IGF-1, VEGF, and HIF-1, among others, were affected by the presence of a functional microbiome (Zhang et al., 2022). Recently, Westway et al. found that infants diagnosed with ROP (stage 1 or greater) had a lower taxonomic microbial diversity than infants with no ROP at admission (Westaway et al., 2022). Furthermore, ROP was signiﬁcantly associated with an enrichment of bacteria from the Gram-positive genus *Staphylococcus*.

Gut microbes interact and communicate with their host through an elaborate crosstalk involving metabolites and other signaling molecules. One such mechanism is through the release of short-chain fatty acids (SCFAs) produced by anaerobic fermentation of human milk oligosaccharides (HMOs) by certain intestinal bacteria. SCFAs from gut microbiota affect host inflammation and glucose and lipid metabolism and contribute to microglia maturation and function in mice (Erny et al., 2015). Levels of SCFAs in stool collected on day 14 and 28 from infants born <28 weeks’ GA were not associated with the risk of any level of ROP (Frazer et al., 2022). However, most of the gut SCFAs are effectively absorbed by colonocytes and used as energy or further transported to the systemic circulation. Thus, quantification of circulatory SFCAs in preterm infants at risk of ROP could shed further light on gut metabolic activity and host interaction in relation to disease development. We conclude that targeting the microbiome and the gut-retina axis through pre- and probiotics may be a new therapeutic avenue in the prevention of ROP.

### Current treatments in practice or in clinical trial

#### Inhibiting neovascularization in Phase II ROP

##### Laser photocoagulation

Current clinical treatments to control neovascularization in Phase II ROP include laser photocoagulation and anti-VEGF therapy. Both therapies target the avascular retina to limit the production of factors induced by hypoxia and fuel insufficiency that cause pathological retinal neovascularization. Laser therapy ablates the more avascular peripheral retina but also causes permanent destruction of the peripheral retina (Clark and Mandal, 2008).

##### Anti-VEGF treatment

VEGF is a key growth factor controlling vascular and neuronal development. Increased levels of hypoxia-induced VEGF are a significant factor contributing to the neovascularization of Phase II ROP. Anti-VEGF therapy which quickly suppresses neovascularization avoids some of the adverse effects of laser therapy such as retinal scarring but also has its own adverse effects. Inhibition of VEGF using Bevacizumab, Ranibizumab, Conbercept and Aflibercept show efficacy in ROP (Table 2; Cheng et al., 2018; Mintz-Hittner et al., 2011; Stahl et al., 2018; Wallace et al., 2018). The BEAT-ROP trial found that intravitreal bevacizumab (0.625 mg or 50% of the adult dose) monotherapy is effective in preventing ROP progression (Mintz-Hittner et al., 2011). However, intravitreal injections of anti-VEGF drugs leak into the systemic circulation lasting up to 2 months after a single injection (Kong et al., 2015; Sato et al., 2012; Hartnett et al., 2022; Wu et al., 2015). The persistent anti-VEGF effect can suppress physiological vascular growth locally in the eye, but also systemically, creating safety concerns in the developing preterm infant. There may be increased neurological damage in preterm infants with the use of anti-VEGF drugs (Arima et al., 2020). Neural retinal damage persists even after the vascular pathology has resolved (Hansen et al., 2017). Efforts have been made to determine a minimal dose to maintain durable suppression of retinal neovascularization while avoiding suppression of plasma VEGF (Cheng et al., 2018; Stahl et al., 2018). Intravitreal Bevacizumab (as low as 0.031 mg) can result in good retinal structural outcomes. However, multiple treatments are needed in many eyes (Wallace et al., 2018). Intravitreal ranibizumab is effective in controlling acute ROP at low doses (0.12 mg and 0.2 mg). Superior vascularization of the peripheral retina is found with 0.12 mg of ranibizumab (Stahl et al., 2018). Intravitreal Conbercept at a low dose (0.15 mg) is effective for Zone II Stage 2/3+RIO and no adverse ocular outcomes were observed during the follow-up period until 90 weeks postmenstrual age (Cheng et al., 2018). A recent Phase 3 trial of intravitreal Aflibercept (0.4 mg) versus laser therapy showed that treatment success is similar between the two groups and less rescue treatment is required in the Aflibercept-treated group (Stahl et al., 2022). However, the drug persists in systemic circulation for at least 8 weeks post intravitreal injection.

**Table 2.** Summary of current or potential treatments for Phase II ROP.

Drug & dose	Sample size	Outcomes	References
***Anti-VEGF (intravitreal)***			
Bevacizumab (0.625 mg)	150 infants (BW <1500 g, GA <30 weeks)	Benefits zone I not zone II posterior stage 3+ROP	Mintz-Hittner et al, N Engl J Med. 2011 Feb 17;364(7):603–15.
Bevacizumab (0.25 mg, 0.125 mg, 0.063 mg, 0.031 mg)	61 infants (mean BW = 709 g, mean GA = 24.9 weeks)	ROP regression by 6 months corrected age and very good retinal structure	Wallace et al, Ophthalmology. 2018 December; 125(12): 1961–1966.
Ranibizumab (0.12 mg and 0.2 mg)	19 infants (mean GA = 36.4 weeks)	Required no rescue therapy; systemic VEGF levels not suppressed.	Stahl et al, JAMA Pediatr. 2018 Mar 1;172(3):278–286.
Conbercept (0.15 mg)	20 infants (mean BW = 1297.5 g, mean GA = 28.6 weeks)	Complete regression of retinopathy and retinal vascularization to zone III	Cheng et al, Sci Rep. 2018 Jul 16;8 (1):10732.
Aflibercept (0.4 mg)	118 infants (GA <32 weeks)	Rescue treatment required in 4.8% aflibercept group vs 11.1% with laser. Serious adverse event rates were similar.	Stahl et al, JAMA. 2022 Jul 26;328(4):348–359.
***Dexamethasone***			
Eye drop (1 mg/ml, 1 drop daily)	48 premature infants	Reduced laser ablation	Öhnell et al, Ophthalmol Retina. 2022 Feb;6 (2):181–182.
Antenatal systemic dexamethasone	63 infants (mean BW = 981 g, mean GA = 27.8 weeks)	Decreased incidence of ROP of stage 2 or higher	Higgins et al, Arch Ophthalmol. 1998 May;116(5):601–5.
≤1.8 mg/kg (low cumulative) or >1.8 mg/kg (high cumulative) body weight (via bolus intravenous infusion)	115 infants (BW ≤1250 g, GA ≤32 weeks)	No association between dexamethasone and severe ROP incidence	Cuculich et al, Biol Neonate. 2001 Jan;79(1):9–14.
0–0.9 mg/kg or 0–0.73 mg/kg (accumulative, systemic)	74 infants (GA <28 weeks)	Higher dose was associated with severe ROP	Pediatr Neonatol. 2022 May;63(3):220–226
***Platelets***			
Low Platelets	202 preterm infants (GA <34 weeks)	Less incidence of ROP with higher platelet count	Cakir et al, JCI Insight. 2018 Oct 4;3 (19):e99448
Platelet transfusion	136 infants (Mean GA = 25.3 weeks, mean BW = 782 g)	Less incidence of ROP	Faheem et al, Annals of R.S.C.B., 2021, 25 (6): 5442–5448

##### Steroid treatment for ROP

In addition to VEGF, inflammatory mediators such as tumor necrosis factor-alpha (TNFα) are also involved in the development and progression of ROP (Connor et al., 2007). Dexamethasone greatly decreases retinal *Tnfα* expression in mice with hypoxia-induced retinopathy (Yossuck et al., 2001). Pretreatment with dexamethasone (0.5 mg/kg subcutaneously) before a high oxygen challenge reduces retinal neovascularization in mouse OIR (Yossuck et al., 2000). An observational cohort study reported that antenatal dexamethasone administration in mothers seems to be associated with a decreased incidence of ROP (stage 2 or higher) in preterm infants with very-low birth weight and low gestational age (Higgins et al., 1998). A recent retrospective study examining premature infants who received topical dexamethasone eye drops before potential laser treatment found that fewer progressed to severe ROP requiring treatment. (Öhnell et al., 2022). Currently, a clinical trial titled “Pharmacokinetics and Safety of Dexamethasone Eye Drops in Preterm Infants” (ClinicalTrials.gov Identifier: NCT05387941) is investigating efficacy of topical dexamethasone treatment before florid neovascularization to prevent progression. An experimental study shows that systemic dexamethasone treatment (0.5 mg/kg subcutaneously) before but not after high oxygen exposure inhibits retinal neovascularization (Yossuck et al., 2000), suggesting that the timing of treatment is critical. The outcomes of dexamethasone treatment appear to be influenced by the dose and route of administration (Cuculich et al., 2001; Tao, 2022). Further optimization of the dose, route and timing of administration is needed.

##### Platelets and ROP

An experimental (and clinical) study found that platelet depletion increases and platelet transfusion decreases hypoxia-induced retinal neovascularization in OIR mice (Cakir et al., 2018). In the same study, retinal VEGF-A expression was found to be induced with platelet depletion and decreased with platelet transfusion and that clinically, platelet deficiency is associated with severe ROP (Cakir et al., 2018). Other studies have also shown this association (Jensen et al., 2018; Parrozzani et al., 2021; Şahinoğlu Keşkek et al., 2020; Seliniotaki et al., 2022). A hospital based prospective study found that platelet transfusion protects against ROP development (Faheem et al., 2021). A correlation was found between platelet count and serum VEGF-A, platelet-derived growth factor (PDGF-BB), and brain-derived neurotrophic factor (BDNF) from serum samples taken on the same day in preterm infants (Hellgren et al., 2021). Taken together, platelet transfusion may be a promising therapeutic approach for ROP prevention and treatment.

### Preventing vessel loss in Phase I ROP

#### Oxygen control

Oxygen supplementation is commonly used to treat preterm infants with poor lung development to increase their survival. However, the correct balance between high oxygen supplementation to decrease mortality and lower oxygen to prevent Phase I ROP remains unknown (Askie et al., 2011). Several studies examined the risk of ROP and the survival rate with lower vs. higher SpO_2_ with target ranges of: (70–90% vs. 88–98%; Tin et al., 2001), (≤92% vs.>92%; Anderson et al., 2004), (83–90% vs. >90–98%; Chow et al., 2003). The Neonatal Oxygenation Prospective Meta-analysis (NeOProM) Collaboration with about 5000 preterm infants (GA <28 weeks) given oxygen supplementation during the entire postnatal period showed that there is increased mortality associated with a lower SpO_2_ target range (85–89% vs. 91–95%). There was no significance difference in the primary outcome disability including bilateral blindness reported (Askie et al., 2018). However, in this study, timing of different SpO_2_ ranges for Phase I and Phase II was not evaluated. A recent study comparing biphasic (85–92% when GA <34 weeks, 95% when GA ≥34 weeks) vs. static (91–95%) SpO_2_ showed that biphasic SpO_2_ decreases incidence and severity of ROP without increasing mortality (Shukla et al., 2019). This suggests that a biphasic approach of oxygen supplementation may optimize the balance between ROP and mortality.

#### IGF-1

A recent clinical trial of IGF-1 supplementation during early life (rhIGF-1/rhIGFBP-3, 250 mcg/kg/24 hours, continuous intravenous infusion from <24 hr of birth to postmenstrual age 29 weeks, ClinicalTrials.gov Identifier: NCT01096784) found decreased severe bronchopulmonary dysplasia and less severe intraventricular hemorrhage (Ley et al., 2019). Better understanding of the appropriate IGF-1 dose in ROP prevention could be achieved from the ongoing trial using SHP607 (recombinant protein complex of IGF-1/IGFBP3, continuous intravenous infusion of SHP607 250 mcg/kg/24 hr and 400 mcg/kg/24 hours from birth to postmenstrual age 29 weeks+6 days, ClinicalTrials.gov Identifier: NCT03253263).

#### Lipid supplementation

Supplementation of nutrients lacking after premature birth also protects against the incidence and severity of ROP. Both DHA and ARA are considered conditionally essential fatty acids for preterm infants and increasing evidence points to health benefits with their supplementation. Low blood DHA and ARA levels in premature infants correlate with ROP progression (Fu et al., 2015a; Lapillonne et al., 2010; Löfqvist et al., 2018). The Mega Donna Mega trial with enteral intake of DHA (50 mg/kg/day) and ARA (100 mg/kg/day) provided within 3 days after birth until postmenstrual age 40 weeks versus no supplementation increases circulating DHA and ARA levels and reduces severe ROP by 50% (Hellström et al., 2021a). The double-blind parallel clinical trial with enteral DHA supplementation at 75 mg/kg/day versus high oleic sunflower oil to preterm infants for 14 days lowers the incidence of stage 3 ROP (Bernabe-García et al., 2019). The DIAMOND (DHA Intake And Measurement Of Neural Development, ClinicalTrials.gov Identifier: NCT00753818) study show that supplementing DHA:ARA at 1:2 ratio in formula to healthy, term infants from the first two weeks of birth improves visual acuity at one year of age. However, further increasing DHA does not generate additional visual improvement (Birch et al., 2010). Meta-analysis of dietary DHA-supplemented formula vs. DHA-free formula to preterm infants improves visual acuity at 2 and 4 months of corrected age (SanGiovanni et al., 2000).

However, the results of DHA supplementation to prevent ROP in preterm infants are not always consistent and parenteral supplementation of lipids may differ from enteral administration (Nilsson et al., 2019). Intravenous (parenteral) fat emulsion containing fish oil versus soybean and olive oil reduces severe ROP requiring laser therapy in very-low-birth-weight infants (Beken et al., 2014; Pawlik et al., 2011; Pawlik et al., 2014). But in another study, a parenteral lipid emulsion containing fish oil (SMOFlipid) versus olive oil-based (Clinoleic) emulsion only marginally increases circulating DHA levels, reduces ARA levels and has no significant impact on the incidence and severity of ROP (Najm et al., 2017). The outcomes may have been affected by the different period of lipid emulsion delivery ranging from 2 to 28 days (Najm et al., 2017) and by reduced ARA levels, as low postnatal ARA levels strongly predict ROP development (Löfqvist et al., 2018). Further investigation of lipid components, route and time of supplementation, as well as the sex differences should be considered to optimize the nutrient supply for best clinical outcomes.

### Tools for future retinal metabolic studies

Advanced technologies make it feasible to investigate detailed metabolic status in the eye. Single-cell transcriptomics and spatial transcriptome profiling are useful in preclinical retinal studies. Metabolomics, lipidomics, and proteomics of blood and retinas have been applied to clinical and experimental studies to detect metabolic biomarkers for ROP. The development and application of these ‘omics’ approaches at the single cell level (Li et al., 2021; Perkel, 2021; Seydel, 2021) should expand our understanding of cell-specific activity. Although Seahorse XF analysis measuring oxygen consumption rate and extracellular acidification rate in a closed and rapidly depleted system has been used for metabolic investigation in cell culture, in tissue samples this technique is restricted by the limited nutrient and oxygen supply which affects readout. A microfluidics flow system providing a continuous supply of nutrients and oxygen, to maintain tissue viability and functionality for a much longer period of time has added to our understanding of retinal metabolism (Bisbach et al., 2020; Rountree et al., 2016; Tsantilas et al., 2021). Organ-on-a-chip technology which can evaluate human tissue and can be used to assess physical and biochemical stimuli, may be a better system than conventional 3D cell culture systems in vitro.

### Conclusion

In summary, our current knowledge of the impact of metabolic disruption such as lipid deficiency, starvation and hyperglycemia on the immature retina in preterm infants is limited. Improving retinal development at an early stage may help prevent ROP before progression to vision-threatening neovascularization. A better understanding of substrate use and metabolic shifts in the neonatal period will help determine how to promote retinal neuronal and vascular maturation by supplementing proper nutrients under hyperglycemic, hyperoxic, and hypoxic conditions. Metabolic modulation to normalize concentrations of naturally occurring growth factors like IGF-1, APN and FGF21 might also be more physiological interventions. Compared to pharmaceutical interventions, modulation and normalization of nutrient supplementation is relatively safe for fragile premature infants. Understanding the correlation between nutrient shortage after premature birth and retinal development will help find effective approaches for disease prevention at an early stage.

## References

[bib1] Adler L t., Chen C, Koutalos Y (2014). Mitochondria contribute to NADPH generation in mouse rod photoreceptors. The Journal of Biological Chemistry.

[bib2] Ahmed J, Pulfer MK, Linsenmeier RA (2001). Measurement of blood flow through the retinal circulation of the cat during normoxia and hypoxemia using fluorescent microspheres. Microvascular Research.

[bib3] Akula JD, Hansen RM, Martinez-Perez ME, Fulton AB (2007). Rod photoreceptor function predicts blood vessel abnormality in retinopathy of prematurity. Investigative Ophthalmology & Visual Science.

[bib4] Anand-Apte B, Hollyfield JG (2010). Developmental anatomy of the retinal and choroidal vasculature. Encyclopedia of the Eye.

[bib5] Anderson CG, Benitz WE, Madan A (2004). Retinopathy of prematurity and pulse oximetry: a national survey of recent practices. Journal of Perinatology.

[bib6] Arboleya S, Sánchez B, Milani C, Duranti S, Solís G, Fernández N, de los Reyes-Gavilán CG, Ventura M, Margolles A, Gueimonde M (2015). Intestinal microbiota development in preterm neonates and effect of perinatal antibiotics. The Journal of Pediatrics.

[bib7] Arima M, Akiyama M, Fujiwara K, Mori Y, Inoue H, Seki E, Nakama T, Tsukamoto S, Ochiai M, Ohga S, Sonoda K-H (2020). Neurodevelopmental outcomes following intravitreal bevacizumab injection in Japanese preterm infants with type 1 retinopathy of prematurity. PLOS ONE.

[bib8] Arnold T, Betsholtz C (2013). The importance of microglia in the development of the vasculature in the central nervous system. Vascular Cell.

[bib9] Askie LM, Brocklehurst P, Darlow BA, Finer N, Schmidt B, Tarnow-Mordi W, Ne OCG (2011). NeOProM: neonatal oxygenation prospective meta-analysis collaboration study protocol. BMC Pediatrics.

[bib10] Askie LM, Darlow BA, Finer N, Schmidt B, Stenson B, Tarnow-Mordi W, Davis PG, Carlo WA, Brocklehurst P, Davies LC, Das A, Rich W, Gantz MG, Roberts RS, Whyte RK, Costantini L, Poets C, Asztalos E, Battin M, Halliday HL, Marlow N, Tin W, King A, Juszczak E, Morley CJ, Doyle LW, Gebski V, Hunter KE, Simes RJ, Neonatal Oxygenation Prospective Meta-analysis Collaboration (2018). Association between oxygen saturation targeting and death or disability in extremely preterm infants in the neonatal oxygenation prospective meta-analysis collaboration. JAMA.

[bib11] Ball JM, Chen S, Li W (2022). Mitochondria in cone photoreceptors act as microlenses to enhance photon delivery and confer directional sensitivity to light. Science Advances.

[bib12] Banjac L, Banjac G, Kotur-Stevuljević J, Spasojević-Kalimanovska V, Gojković T, Bogavac-Stanojević N, Jelić-Ivanović Z, Banjac G (2018). Pro-Oxidants and antioxidants in retinopathy of prematurity. Acta Clinica Croatica.

[bib13] Beardsall K, Vanhaesebrouck S, Ogilvy-Stuart AL, Vanhole C, Palmer CR, van Weissenbruch M, Midgley P, Thompson M, Thio M, Cornette L, Ossuetta I, Iglesias I, Theyskens C, de Jong M, Ahluwalia JS, de Zegher F, Dunger DB (2008). Early insulin therapy in very-low-birth-weight infants. The New England Journal of Medicine.

[bib14] Beauchamp MH, Martinez-Bermudez AK, Gobeil F, Marrache AM, Hou X, Speranza G, Abran D, Quiniou C, Lachapelle P, Roberts J, Almazan G, Varma DR, Chemtob S (2001). Role of thromboxane in retinal microvascular degeneration in oxygen-induced retinopathy. Journal of Applied Physiology.

[bib15] Becker S, Wang H, Simmons AB, Suwanmanee T, Stoddard GJ, Kafri T, Hartnett ME (2018). Targeted knockdown of overexpressed VEGFA or VEGF164 in Müller cells maintains retinal function by triggering different signaling mechanisms. Scientific Reports.

[bib16] Beken S, Dilli D, Fettah ND, Kabataş EU, Zenciroğlu A, Okumuş N (2014). The influence of fish-oil lipid emulsions on retinopathy of prematurity in very low birth weight infants: a randomized controlled trial. Early Human Development.

[bib17] Bernabe-García M, Villegas-Silva R, Villavicencio-Torres A, Calder PC, Rodríguez-Cruz M, Maldonado-Hernández J, Macías-Loaiza D, López-Alarcón M, Inda-Icaza P, Cruz-Reynoso L (2019). Enteral docosahexaenoic acid and retinopathy of prematurity: a randomized clinical trial. JPEN. Journal of Parenteral and Enteral Nutrition.

[bib18] Binder ND, Raschko PK, Benda GI, Reynolds JW (1989). Insulin infusion with parenteral nutrition in extremely low birth weight infants with hyperglycemia. The Journal of Pediatrics.

[bib19] Binet F, Cagnone G, Crespo-Garcia S, Hata M, Neault M, Dejda A, Wilson AM, Buscarlet M, Mawambo GT, Howard JP, Diaz-Marin R, Parinot C, Guber V, Pilon F, Juneau R, Laflamme R, Sawchyn C, Boulay K, Leclerc S, Abu-Thuraia A, Côté J-F, Andelfinger G, Rezende FA, Sennlaub F, Joyal J-S, Mallette FA, Sapieha P (2020). Neutrophil extracellular traps target senescent vasculature for tissue remodeling in retinopathy. Science.

[bib20] Birch EE, Carlson SE, Hoffman DR, Fitzgerald-Gustafson KM, Fu VLN, Drover JR, Castañeda YS, Minns L, Wheaton DKH, Mundy D, Marunycz J, Diersen-Schade DA (2010). The diamond (DHA intake and measurement of neural development) study: a double-masked, randomized controlled clinical trial of the maturation of infant visual acuity as a function of the dietary level of docosahexaenoic acid. The American Journal of Clinical Nutrition.

[bib21] Birtel J, von Landenberg C, Gliem M, Gliem C, Reimann J, Kunz WS, Herrmann P, Betz C, Caswell R, Nesbitt V, Kornblum C, Charbel Issa P (2022). Mitochondrial retinopathy. Ophthalmology. Retina.

[bib22] Bisbach CM, Hass DT, Robbings BM, Rountree AM, Sadilek M, Sweet IR, Hurley JB (2020). Succinate can shuttle reducing power from the hypoxic retina to the o2-rich pigment epithelium. Cell Reports.

[bib23] Blanco CL, Baillargeon JG, Morrison RL, Gong AK (2006). Hyperglycemia in extremely low birth weight infants in a predominantly Hispanic population and related morbidities. Journal of Perinatology.

[bib24] Boeck M, Thien A, Wolf J, Hagemeyer N, Laich Y, Yusuf D, Backofen R, Zhang P, Boneva S, Stahl A, Hilgendorf I, Agostini H, Prinz M, Wieghofer P, Schlunck G, Schlecht A, Lange C (2020). Temporospatial distribution and transcriptional profile of retinal microglia in the oxygen-induced retinopathy mouse model. Glia.

[bib25] Boskabadi H, Marefat M, Maamouri G, Abrishami M, Abrishami M, Shoeibi N, Sanjari MS, Mobarhan MG, Shojaei SRH, Tavallaei S, Sanei F, Kakavandi M, Moradi A, Zakerihamidi M (2021). Evaluation of pro-oxidant antioxidant balance in retinopathy of prematurity. Eye.

[bib26] Brodin P (2022). Immune-microbe interactions early in life: a determinant of health and disease long term. Science.

[bib27] Brooks SE, Gu X, Samuel S, Marcus DM, Bartoli M, Huang PL, Caldwell RB (2001). Reduced severity of oxygen-induced retinopathy in eNOS-deficient mice. Investigative Ophthalmology & Visual Science.

[bib28] Cakir B, Liegl R, Hellgren G, Lundgren P, Sun Y, Klevebro S, Löfqvist C, Mannheimer C, Cho S, Poblete A, Duran R, Hallberg B, Canas J, Lorenz V, Liu ZJ, Sola-Visner MC, Smith LE, Hellström A (2018). Thrombocytopenia is associated with severe retinopathy of prematurity. JCI Insight.

[bib29] Cakir B, Hellström W, Tomita Y, Fu Z, Liegl R, Winberg A, Hansen-Pupp I, Ley D, Hellström A, Löfqvist C, Smith LE (2020). Igf1, serum glucose, and retinopathy of prematurity in extremely preterm infants. JCI Insight.

[bib30] Casson RJ, Wood JPM, Han G, Kittipassorn T, Peet DJ, Chidlow G (2016). M-Type pyruvate kinase isoforms and lactate dehydrogenase A in the mammalian retina: metabolic implications. Investigative Ophthalmology & Visual Science.

[bib31] Chan-Ling T, Gock B, Stone J (1995). The effect of oxygen on vasoformative cell division. Evidence that “ physiological hypoxia ” is the stimulus for normal retinal vasculogenesis. Investigative Ophthalmology & Visual Science.

[bib32] Chavez-Valdez R, McGowan J, Cannon E, Lehmann CU (2011). Contribution of early glycemic status in the development of severe retinopathy of prematurity in a cohort of ELBW infants. Journal of Perinatology.

[bib33] Chen CT, Shao Z, Fu Z (2022). Dysfunctional peroxisomal lipid metabolisms and their ocular manifestations. Frontiers in Cell and Developmental Biology.

[bib34] Cheng Y, Meng Q, Linghu D, Zhao M, Liang J (2018). A lower dose of intravitreal conbercept effectively treats retinopathy of prematurity. Scientific Reports.

[bib35] Chinchore Y, Begaj T, Wu D, Drokhlyansky E, Cepko CL (2017). Glycolytic reliance promotes anabolism in photoreceptors. eLife.

[bib36] Chow LC, Wright KW, Sola A, CSMC Oxygen Administration Study Group (2003). Can changes in clinical practice decrease the incidence of severe retinopathy of prematurity in very low birth weight infants?. Pediatrics.

[bib37] Cipolla CM, Lodhi IJ (2017). Peroxisomal dysfunction in age-related diseases. Trends in Endocrinology and Metabolism.

[bib38] Clark D, Mandal K (2008). Treatment of retinopathy of prematurity. Early Human Development.

[bib39] Cohen LH, Noell WK (1960). Glucose catabolism of rabbit retina before and after development of visual function. Journal of Neurochemistry.

[bib40] Connor KM, SanGiovanni JP, Lofqvist C, Aderman CM, Chen J, Higuchi A, Hong S, Pravda EA, Majchrzak S, Carper D, Hellstrom A, Kang JX, Chew EY, Salem N, Serhan CN, Smith LEH (2007). Increased dietary intake of omega-3-polyunsaturated fatty acids reduces pathological retinal angiogenesis. Nature Medicine.

[bib41] Country MW (2017). Retinal metabolism: A comparative look at energetics in the retina. Brain Research.

[bib42] Crespo-Garcia S, Tsuruda PR, Dejda A, Ryan RD, Fournier F, Chaney SY, Pilon F, Dogan T, Cagnone G, Patel P, Buscarlet M, Dasgupta S, Girouard G, Rao SR, Wilson AM, O’Brien R, Juneau R, Guber V, Dubrac A, Beausejour C, Armstrong S, Mallette FA, Yohn CB, Joyal J-S, Marquess D, Beltran PJ, Sapieha P (2021). Pathological angiogenesis in retinopathy engages cellular senescence and is amenable to therapeutic elimination via Bcl-xL inhibition. Cell Metabolism.

[bib43] Cringle SJ, Yu DY, Alder VA (1991). Intraretinal oxygen tension in the rat eye. Graefe’s Archive for Clinical and Experimental Ophthalmology = Albrecht von Graefes Archiv Fur Klinische Und Experimentelle Ophthalmologie.

[bib44] Cringle SJ, Yu DY (2018). Regulation of oxygen tension in the mammalian retina during systemic hyperoxia is species dependent. Advances in Experimental Medicine and Biology.

[bib45] Cuculich PS, DeLozier KA, Mellen BG, Shenai JP (2001). Postnatal dexamethasone treatment and retinopathy of prematurity in very-low-birth-weight neonates. Biology of the Neonate.

[bib46] Dahlgren AF, Pan A, Lam V, Gouthro KC, Simpson PM, Salzman NH, Nghiem-Rao TH (2019). Longitudinal changes in the gut microbiome of infants on total parenteral nutrition. Pediatric Research.

[bib47] Danielsson H, Tebani A, Zhong W, Fagerberg L, Brusselaers N, Hård A-L, Uhlén M, Hellström A (2022). Blood protein profiles related to preterm birth and retinopathy of prematurity. Pediatric Research.

[bib48] Daruich A, Matet A, Borruat FX (2014). Macular dystrophy associated with the mitochondrial DNA A3243G mutation: pericentral pigment deposits or atrophy? report of two cases and review of the literature. BMC Ophthalmology.

[bib49] Das Y, Swinkels D, Baes M (2021). Peroxisomal disorders and their mouse models point to essential roles of peroxisomes for retinal integrity. International Journal of Molecular Sciences.

[bib50] Daughaday WH, Rotwein P (1989). Insulin-Like growth factors I and II. peptide, messenger ribonucleic acid and gene structures, serum, and tissue concentrations. Endocrine Reviews.

[bib51] Davies MH, Eubanks JP, Powers MR (2006). Microglia and macrophages are increased in response to ischemia-induced retinopathy in the mouse retina. Molecular Vision.

[bib52] De Bock K, Georgiadou M, Schoors S, Kuchnio A, Wong BW, Cantelmo AR, Quaegebeur A, Ghesquière B, Cauwenberghs S, Eelen G, Phng L-K, Betz I, Tembuyser B, Brepoels K, Welti J, Geudens I, Segura I, Cruys B, Bifari F, Decimo I, Blanco R, Wyns S, Vangindertael J, Rocha S, Collins RT, Munck S, Daelemans D, Imamura H, Devlieger R, Rider M, Van Veldhoven PP, Schuit F, Bartrons R, Hofkens J, Fraisl P, Telang S, Deberardinis RJ, Schoonjans L, Vinckier S, Chesney J, Gerhardt H, Dewerchin M, Carmeliet P (2013). Role of PFKFB3-driven glycolysis in vessel sprouting. Cell.

[bib53] Elmasri H, Karaaslan C, Teper Y, Ghelfi E, Weng M, Ince TA, Kozakewich H, Bischoff J, Cataltepe S (2009). Fatty acid binding protein 4 is a target of VEGF and a regulator of cell proliferation in endothelial cells. FASEB Journal.

[bib54] Engvall M, Kawasaki A, Carelli V, Wibom R, Bruhn H, Lesko N, Schober FA, Wredenberg A, Wedell A, Träisk F (2021). Case report: a novel mutation in the mitochondrial MT-ND5 gene is associated with Leber hereditary optic neuropathy (LHON). Frontiers in Neurology.

[bib55] Eperon G, Johnson M, David NJ (1975). The effect of arterial PO2 on relative retinal blood flow in monkeys. Investigative Ophthalmology.

[bib56] Erny D, Hrabě de Angelis AL, Jaitin D, Wieghofer P, Staszewski O, David E, Keren-Shaul H, Mahlakoiv T, Jakobshagen K, Buch T, Schwierzeck V, Utermöhlen O, Chun E, Garrett WS, McCoy KD, Diefenbach A, Staeheli P, Stecher B, Amit I, Prinz M (2015). Host microbiota constantly control maturation and function of microglia in the CNS. Nature Neuroscience.

[bib57] Faheem M, Harish MM, Mekala S, Baliga K, Rajesh SM, Ravikiran SR, Muralikeshava S (2021). Effect of platelet transfusion on retinopathy of prematurity- hospital based prospective study. Annals of the Romanian Society for Cell Biology.

[bib58] Fouhy F, Watkins C, Hill CJ, O’Shea C-A, Nagle B, Dempsey EM, O’Toole PW, Ross RP, Ryan CA, Stanton C (2019). Perinatal factors affect the gut microbiota up to four years after birth. Nature Communications.

[bib59] Fransen M, Nordgren M, Wang B, Apanasets O (2012). Role of peroxisomes in ROS/RNS-metabolism: implications for human disease. Biochimica et Biophysica Acta.

[bib60] Frazer LC, Yakah W, Martin CR (2022). Decreased acetic acid in the stool of preterm infants is associated with an increased risk of bronchopulmonary dysplasia. Nutrients.

[bib61] Fu ZJ, Li S-Y, Kociok N, Wong D, Chung SK, Lo ACY (2012). Aldose reductase deficiency reduced vascular changes in neonatal mouse retina in oxygen-induced retinopathy. Investigative Ophthalmology & Visual Science.

[bib62] Fu Z, Lofqvist CA, Shao Z, Sun Y, Joyal JS, Hurst CG, Cui RZ, Evans LP, Tian K, SanGiovanni JP, Chen J, Ley D, Hansen Pupp I, Hellstrom A, Smith LEH (2015a). Dietary ω-3 polyunsaturated fatty acids decrease retinal neovascularization by adipose-endoplasmic reticulum stress reduction to increase adiponectin. The American Journal of Clinical Nutrition.

[bib63] Fu Z, Nian S, Li SY, Wong D, Chung SK, Lo ACY (2015b). Deficiency of aldose reductase attenuates inner retinal neuronal changes in a mouse model of retinopathy of prematurity. Graefe’s Archive for Clinical and Experimental Ophthalmology = Albrecht von Graefes Archiv Fur Klinische Und Experimentelle Ophthalmologie.

[bib64] Fu Z, Gong Y, Liegl R, Wang Z, Liu C-H, Meng SS, Burnim SB, Saba NJ, Fredrick TW, Morss PC, Hellstrom A, Talukdar S, Smith LEH (2017). FGF21 administration suppresses retinal and choroidal neovascularization in mice. Cell Reports.

[bib65] Fu Z, Löfqvist CA, Liegl R, Wang Z, Sun Y, Gong Y, Liu CH, Meng SS, Burnim SB, Arellano I, Chouinard MT, Duran R, Poblete A, Cho SS, Akula JD, Kinter M, Ley D, Pupp IH, Talukdar S, Hellström A, Smith LE (2018a). Photoreceptor glucose metabolism determines normal retinal vascular growth. EMBO Molecular Medicine.

[bib66] Fu Z, Wang Z, Liu CH, Gong Y, Cakir B, Liegl R, Sun Y, Meng SS, Burnim SB, Arellano I, Moran E, Duran R, Poblete A, Cho SS, Talukdar S, Akula JD, Hellström A, Smith LEH (2018b). Fibroblast growth factor 21 protects photoreceptor function in type 1 diabetic mice. Diabetes.

[bib67] Fu Z, Sun Y, Cakir B, Tomita Y, Huang S, Wang Z, Liu C-H, S Cho S, Britton W, S Kern T, Antonetti DA, Hellström A, E H Smith L (2020). Targeting neurovascular interaction in retinal disorders. International Journal of Molecular Sciences.

[bib68] Fu Z, Yan W, Chen CT, Nilsson AK, Bull E, Allen W, Yang J, Ko M, SanGiovanni JP, Akula JD, Talukdar S, Hellström A, Smith LEH (2022). Omega-3/Omega-6 long-chain fatty acid imbalance in phase I retinopathy of prematurity. Nutrients.

[bib69] Fulton AB, Akula JD, Mocko JA, Hansen RM, Benador IY, Beck SC, Fahl E, Seeliger MW, Moskowitz A, Harris ME (2009). Retinal degenerative and hypoxic ischemic disease. Documenta Ophthalmologica. Advances in Ophthalmology.

[bib70] Gantner ML, Eade K, Wallace M, Handzlik MK, Fallon R, Trombley J, Bonelli R, Giles S, Harkins-Perry S, Heeren TFC, Sauer L, Ideguchi Y, Baldini M, Scheppke L, Dorrell MI, Kitano M, Hart BJ, Cai C, Nagasaki T, Badur MG, Okada M, Woods SM, Egan C, Gillies M, Guymer R, Eichler F, Bahlo M, Fruttiger M, Allikmets R, Bernstein PS, Metallo CM, Friedlander M (2019). Serine and lipid metabolism in macular disease and peripheral neuropathy. The New England Journal of Medicine.

[bib71] Gibson MK, Wang B, Ahmadi S, Burnham CAD, Tarr PI, Warner BB, Dantas G (2016). Developmental dynamics of the preterm infant gut microbiota and antibiotic resistome. Nature Microbiology.

[bib72] Gospe SM, Travis AM, Kolesnikov AV, Klingeborn M, Wang L, Kefalov VJ, Arshavsky VY (2019). Photoreceptors in a mouse model of leigh syndrome are capable of normal light-evoked signaling. The Journal of Biological Chemistry.

[bib73] Grant ZL, Whitehead L, Wong VH, He Z, Yan RY, Miles AR, Benest AV, Bates DO, Prahst C, Bentley K, Bui BV, Symons RC, Coultas L (2020). Blocking endothelial apoptosis revascularizes the retina in a model of ischemic retinopathy. The Journal of Clinical Investigation.

[bib74] Guasti L, Silvennoinen S, Bulstrode NW, Ferretti P, Sankilampi U, Dunkel L (2014). Elevated FGF21 leads to attenuated postnatal linear growth in preterm infants through GH resistance in chondrocytes. The Journal of Clinical Endocrinology and Metabolism.

[bib75] Gusarova GA, Trejo HE, Dada LA, Briva A, Welch LC, Hamanaka RB, Mutlu GM, Chandel NS, Prakriya M, Sznajder JI (2011). Hypoxia leads to na,K-atpase downregulation via ca(2+) release-activated ca(2+) channels and AMPK activation. Molecular and Cellular Biology.

[bib76] Hadley KB, Ryan AS, Forsyth S, Gautier S, Salem N (2016). The essentiality of arachidonic acid in infant development. Nutrients.

[bib77] Han X, Kong J, Hartnett ME, Wang H (2019). Enhancing retinal endothelial glycolysis by inhibiting UCP2 promotes physiologic retinal vascular development in a model of retinopathy of prematurity. Investigative Ophthalmology & Visual Science.

[bib78] Hansen RM, Moskowitz A, Akula JD, Fulton AB (2017). The neural retina in retinopathy of prematurity. Progress in Retinal and Eye Research.

[bib79] Hård A-L, Smith LE, Hellström A (2013). Nutrition, insulin-like growth factor-1 and retinopathy of prematurity. Seminars in Fetal & Neonatal Medicine.

[bib80] Harrison SA, Ruane PJ, Freilich BL, Neff G, Patil R, Behling CA, Hu C, Fong E, de Temple B, Tillman EJ, Rolph TP, Cheng A, Yale K (2021). Efruxifermin in non-alcoholic steatohepatitis: a randomized, double-blind, placebo-controlled, phase 2a trial. Nature Medicine.

[bib81] Hartnett ME, Wallace DK, Dean TW, Li Z, Boente CS, Dosunmu EO, Freedman SF, Golden RP, Kong L, Prakalapakorn SG, Repka MX, Smith LE, Wang H, Kraker RT, Cotter SA, Holmes JM, Writing Committee for the Pediatric Eye Disease Investigator Group (2022). Plasma levels of bevacizumab and vascular endothelial growth factor after low-dose bevacizumab treatment for retinopathy of prematurity in infants. JAMA Ophthalmology.

[bib82] Heckel E, Cagnone G, Agnihotri T, Cakir B, Das A, Kim JS, Kim N, Lavoie G, Situ A, Pundir S, Sun Y, Wünnemann F, Pierce KA, Dennis C, Mitchell GA, Chemtob S, Rezende FA, Andelfinger G, Clish CB, Roux PP, Sapieha P, Smith LE, Joyal J-S (2022). Triglyceride-derived fatty acids reduce autophagy in a model of retinal angiomatous proliferation. JCI Insight.

[bib83] Hellgren G, Lundgren P, Pivodic A, Löfqvist C, Nilsson AK, Ley D, Sävman K, Smith LE, Hellström A (2021). Decreased platelet counts and serum levels of VEGF-A, PDGF-BB, and BDNF in extremely preterm infants developing severe ROP. Neonatology.

[bib84] Hellstrom A, Perruzzi C, Ju M, Engstrom E, Hard AL, Liu JL, Albertsson-Wikland K, Carlsson B, Niklasson A, Sjodell L, LeRoith D, Senger DR, Smith LE (2001). Low IGF-I suppresses VEGF-survival signaling in retinal endothelial cells: direct correlation with clinical retinopathy of prematurity. PNAS.

[bib85] Hellström A, Engström E, Hård A-L, Albertsson-Wikland K, Carlsson B, Niklasson A, Löfqvist C, Svensson E, Holm S, Ewald U, Holmström G, Smith LEH (2003). Postnatal serum insulin-like growth factor I deficiency is associated with retinopathy of prematurity and other complications of premature birth. Pediatrics.

[bib86] Hellstrom A, Hard AL, Engstrom E, Niklasson A, Andersson E, Smith L, Lofqvist C (2009). Early weight gain predicts retinopathy in preterm infants: new, simple, efficient approach to screening. Pediatrics.

[bib87] Hellström A, Nilsson AK, Wackernagel D, Pivodic A, Vanpee M, Sjöbom U, Hellgren G, Hallberg B, Domellöf M, Klevebro S, Hellström W, Andersson M, Lund AM, Löfqvist C, Elfvin A, Sävman K, Hansen-Pupp I, Hård AL, Smith LEH, Ley D (2021a). Effect of enteral lipid supplement on severe retinopathy of prematurity: A randomized clinical trial. JAMA Pediatrics.

[bib88] Hellström A, Pivodic A, Gränse L, Lundgren P, Sjöbom U, Nilsson AK, Söderling H, Hård AL, Smith LEH, Löfqvist CA (2021b). Association of docosahexaenoic acid and arachidonic acid serum levels with retinopathy of prematurity in preterm infants. JAMA Network Open.

[bib89] Higgins RD, Mendelsohn AL, DeFeo MJ, Ucsel R, Hendricks-Munoz KD (1998). Antenatal dexamethasone and decreased severity of retinopathy of prematurity. Archives of Ophthalmology.

[bib90] Holland WL, Adams AC, Brozinick JT, Bui HH, Miyauchi Y, Kusminski CM, Bauer SM, Wade M, Singhal E, Cheng CC, Volk K, Kuo M-S, Gordillo R, Kharitonenkov A, Scherer PE (2013). An FGF21-adiponectin-ceramide axis controls energy expenditure and insulin action in mice. Cell Metabolism.

[bib91] Huang H, Vandekeere S, Kalucka J, Bierhansl L, Zecchin A, Brüning U, Visnagri A, Yuldasheva N, Goveia J, Cruys B, Brepoels K, Wyns S, Rayport S, Ghesquière B, Vinckier S, Schoonjans L, Cubbon R, Dewerchin M, Eelen G, Carmeliet P (2017). Role of glutamine and interlinked asparagine metabolism in vessel formation. The EMBO Journal.

[bib92] Hurley JB (2021). Retina metabolism and metabolism in the pigmented epithelium: a busy intersection. Annual Review of Vision Science.

[bib93] Hutter D, Kingdom J, Jaeggi E (2010). Causes and mechanisms of intrauterine hypoxia and its impact on the fetal cardiovascular system: a review. International Journal of Pediatrics.

[bib94] Hutto RA, Bisbach CM, Abbas F, Brock DC, Cleghorn WM, Parker ED, Bauer BH, Ge W, Vinberg F, Hurley JB, Brockerhoff SE (2020). Increasing Ca2+ in photoreceptor mitochondria alters metabolites, accelerates photoresponse recovery, and reveals adaptations to mitochondrial stress. Cell Death and Differentiation.

[bib95] Jensen AK, Ying GS, Huang J, Quinn GE, Binenbaum G (2017). Postnatal serum insulin-like growth factor I and retinopathy of prematurity. Retina.

[bib96] Jensen AK, Ying GS, Huang J, Quinn GE, Binenbaum G (2018). Longitudinal study of the association between thrombocytopenia and retinopathy of prematurity. Journal of AAPOS.

[bib97] Jiang F, Wang Y, Du S, Jin H, Han J (2020). Rapamycin prevents retinal neovascularization by downregulation of cyclin D1 in a mouse model of oxygen-induced retinopathy. BMC Ophthalmology.

[bib98] Joyal J-S, Sitaras N, Binet F, Rivera JC, Stahl A, Zaniolo K, Shao Z, Polosa A, Zhu T, Hamel D, Djavari M, Kunik D, Honoré J-C, Picard E, Zabeida A, Varma DR, Hickson G, Mancini J, Klagsbrun M, Costantino S, Beauséjour C, Lachapelle P, Smith LEH, Chemtob S, Sapieha P (2011). Ischemic neurons prevent vascular regeneration of neural tissue by secreting semaphorin 3A. Blood.

[bib99] Joyal J-S, Sun Y, Gantner ML, Shao Z, Evans LP, Saba N, Fredrick T, Burnim S, Kim JS, Patel G, Juan AM, Hurst CG, Hatton CJ, Cui Z, Pierce KA, Bherer P, Aguilar E, Powner MB, Vevis K, Boisvert M, Fu Z, Levy E, Fruttiger M, Packard A, Rezende FA, Maranda B, Sapieha P, Chen J, Friedlander M, Clish CB, Smith LEH (2016). Retinal lipid and glucose metabolism dictates angiogenesis through the lipid sensor FFAR1. Nature Medicine.

[bib100] Joyal JS, Gantner ML, Smith LEH (2018). Retinal energy demands control vascular supply of the retina in development and disease: the role of neuronal lipid and glucose metabolism. Progress in Retinal and Eye Research.

[bib101] Jung F, Palmer LA, Zhou N, Johns RA (2000). Hypoxic regulation of inducible nitric oxide synthase via hypoxia inducible factor-1 in cardiac myocytes. Circulation Research.

[bib102] Kaempf JW, Kaempf AJ, Wu Y, Stawarz M, Niemeyer J, Grunkemeier G (2011). Hyperglycemia, insulin and slower growth velocity may increase the risk of retinopathy of prematurity. Journal of Perinatology.

[bib103] Kanow MA, Giarmarco MM, Jankowski CS, Tsantilas K, Engel AL, Du J, Linton JD, Farnsworth CC, Sloat SR, Rountree A, Sweet IR, Lindsay KJ, Parker ED, Brockerhoff SE, Sadilek M, Chao JR, Hurley JB (2017). Biochemical adaptations of the retina and retinal pigment epithelium support a metabolic ecosystem in the vertebrate eye. eLife.

[bib104] Kaufman A, Abuqayyas L, Denney WS, Tillman EJ, Rolph T (2020). AKR-001, an fc-FGF21 analog, showed sustained pharmacodynamic effects on insulin sensitivity and lipid metabolism in type 2 diabetes patients. Cell Reports. Medicine.

[bib105] Kermorvant-Duchemin E, Pinel AC, Lavalette S, Lenne D, Raoul W, Calippe B, Behar-Cohen F, Sahel J-A, Guillonneau X, Sennlaub F (2013). Neonatal hyperglycemia inhibits angiogenesis and induces inflammation and neuronal degeneration in the retina. PLOS ONE.

[bib106] Kierans SJ, Taylor CT (2021). Regulation of glycolysis by the hypoxia-inducible factor (HIF): implications for cellular physiology. The Journal of Physiology.

[bib107] Kim J, Tchernyshyov I, Semenza GL, Dang CV (2006). Hif-1-Mediated expression of pyruvate dehydrogenase kinase: a metabolic switch required for cellular adaptation to hypoxia. Cell Metabolism.

[bib108] Kim Y, Hong HK, Park JR, Choi W, Woo SJ, Park KH, Oh WY (2018). Oxygen-Induced retinopathy and choroidopathy: in vivo longitudinal observation of vascular changes using OCTA. Investigative Ophthalmology & Visual Science.

[bib109] Kim SA, Kim SJ, Choi YA, Yoon HJ, Kim A, Lee J (2020). Retinal VEGFA maintains the ultrastructure and function of choriocapillaris by preserving the endothelial PLVAP. Biochemical and Biophysical Research Communications.

[bib110] Kong L, Bhatt AR, Demny AB, Coats DK, Li A, Rahman EZ, Smith OE, Steinkuller PG (2015). Pharmacokinetics of bevacizumab and its effects on serum VEGF and IGF-1 in infants with retinopathy of prematurity. Investigative Ophthalmology & Visual Science.

[bib111] Kumar A, Ranjan R, Basu S, Khanna HD, Bhargava V (2008). Antioxidant levels in cord blood of low birth weight newborns. Indian Pediatrics.

[bib112] Kumbhare SV, Jones WD, Fast S, Bonner C, Van Domselaar G, Graham M, Narvey M, Azad MB (2022). Source of human milk (mother or donor) is more important than fortifier type (human or bovine) in shaping the preterm infant microbiome. Cell Reports. Medicine.

[bib113] Lange J, Yafai Y, Noack A, Yang XM, Munk A-B, Krohn S, Iandiev I, Wiedemann P, Reichenbach A, Eichler W (2012). The axon guidance molecule netrin-4 is expressed by müller cells and contributes to angiogenesis in the retina. Glia.

[bib114] Lapillonne A, Eleni dit Trolli S, Kermorvant-Duchemin E (2010). Postnatal docosahexaenoic acid deficiency is an inevitable consequence of current recommendations and practice in preterm infants. Neonatology.

[bib115] Lara-Cantón I, Badurdeen S, Dekker J, Davis P, Roberts C, Te Pas A, Vento M (2022). Oxygen saturation and heart rate in healthy term and late preterm infants with delayed cord clamping. Pediatric Research.

[bib116] Le YZ (2017). Vegf production and signaling in Müller glia are critical to modulating vascular function and neuronal integrity in diabetic retinopathy and hypoxic retinal vascular diseases. Vision Research.

[bib117] Lee JH, Hornik CP, Testoni D, Laughon MM, Cotten CM, Maldonado RS, Belcastro MR, Clark RH, Smith PB (2016). Insulin, hyperglycemia, and severe retinopathy of prematurity in extremely low-birth-weight infants. American Journal of Perinatology.

[bib118] Ley D, Hallberg B, Hansen-Pupp I, Dani C, Ramenghi LA, Marlow N, Beardsall K, Bhatti F, Dunger D, Higginson JD, Mahaveer A, Mezu-Ndubuisi OJ, Reynolds P, Giannantonio C, van Weissenbruch M, Barton N, Tocoian A, Hamdani M, Jochim E, Mangili A, Chung J-K, Turner MA, Smith LEH, Hellström A, study team (2019). RhIGF-1/rhigfbp-3 in preterm infants: a phase 2 randomized controlled trial. The Journal of Pediatrics.

[bib119] Li Z, Cheng S, Lin Q, Cao W, Yang J, Zhang M, Shen A, Zhang W, Xia Y, Ma X, Ouyang Z (2021). Single-Cell lipidomics with high structural specificity by mass spectrometry. Nature Communications.

[bib120] Liegl R, Löfqvist C, Hellström A, Smith LEH (2016). Igf-1 in retinopathy of prematurity, a CNS neurovascular disease. Early Human Development.

[bib121] Lindsay KJ, Du J, Sloat SR, Contreras L, Linton JD, Turner SJ, Sadilek M, Satrústegui J, Hurley JB (2014). Pyruvate kinase and aspartate-glutamate carrier distributions reveal key metabolic links between neurons and glia in retina. PNAS.

[bib122] Linsenmeier RA (1986). Effects of light and darkness on oxygen distribution and consumption in the cat retina. The Journal of General Physiology.

[bib123] Linsenmeier RA, Yancey CM (1989). Effects of hyperoxia on the oxygen distribution in the intact cat retina. Investigative Ophthalmology & Visual Science.

[bib124] Linsenmeier RA, Braun RD (1992). Oxygen distribution and consumption in the cat retina during normoxia and hypoxemia. The Journal of General Physiology.

[bib125] Linsenmeier RA, Zhang HF (2017). Retinal oxygen: from animals to humans. Progress in Retinal and Eye Research.

[bib126] Liu Z, Yan S, Wang J, Xu Y, Wang Y, Zhang S, Xu X, Yang Q, Zeng X, Zhou Y, Gu X, Lu S, Fu Z, Fulton DJ, Weintraub NL, Caldwell RB, Zhang W, Wu C, Liu X-L, Chen J-F, Ahmad A, Kaddour-Djebbar I, Al-Shabrawey M, Li Q, Jiang X, Sun Y, Sodhi A, Smith L, Hong M, Huo Y (2017). Endothelial adenosine A2A receptor-mediated glycolysis is essential for pathological retinal angiogenesis. Nature Communications.

[bib127] Liu J, Tsang JKW, Fung FKC, Chung SK, Fu Z, Lo ACY (2022). Retinal microglia protect against vascular damage in a mouse model of retinopathy of prematurity. Frontiers in Pharmacology.

[bib128] Löfqvist C, Hansen-Pupp I, Andersson E, Holm K, Smith LEH, Ley D, Hellström A (2009). Validation of a new retinopathy of prematurity screening method monitoring longitudinal postnatal weight and insulinlike growth factor I. Archives of Ophthalmology.

[bib129] Löfqvist CA, Najm S, Hellgren G, Engström E, Sävman K, Nilsson AK, Andersson MX, Hård A-L, Smith LEH, Hellström A (2018). Association of retinopathy of prematurity with low levels of arachidonic acid: a secondary analysis of a randomized clinical trial. JAMA Ophthalmology.

[bib130] Lynch AM, Wagner BD, Mandava N, Palestine AG, Mourani PM, McCourt EA, Oliver SCN, Abman SH (2016). The relationship of novel plasma proteins in the early neonatal period with retinopathy of prematurity. Investigative Ophthalmology & Visual Science.

[bib131] Madaan A, Chaudhari P, Nadeau-Vallée M, Hamel D, Zhu T, Mitchell G, Samuels M, Pundir S, Dabouz R, Howe Cheng CW, Mohammad Nezhady MA, Joyal J-S, Rivera JC, Chemtob S (2019). Müller cell-localized G-protein-coupled receptor 81 (hydroxycarboxylic acid receptor 1) regulates inner retinal vasculature via norrin/wnt pathways. The American Journal of Pathology.

[bib132] Mericq V, De Luca F, Hernandez MI, Peña V, Rossel K, Garcia M, Avila A, Cavada G, Iñiguez G (2014). Serum fibroblast growth factor 21 levels are inversely associated with growth rates in infancy. Hormone Research in Paediatrics.

[bib133] Mintz-Hittner HA, Kennedy KA, Chuang AZ, Group BRC (2011). Efficacy of intravitreal bevacizumab for stage 3+ retinopathy of prematurity. The New England Journal of Medicine.

[bib134] Mobasheri A, Richardson S, Mobasheri R, Shakibaei M, Hoyland JA (2005). Hypoxia inducible factor-1 and facilitative glucose transporters GLUT1 and GLUT3: putative molecular components of the oxygen and glucose sensing apparatus in articular chondrocytes. Histology and Histopathology.

[bib135] Mohsen L, Abou-Alam M, El-Dib M, Labib M, Elsada M, Aly H (2014). A prospective study on hyperglycemia and retinopathy of prematurity. Journal of Perinatology.

[bib136] Najm S, Löfqvist C, Hellgren G, Engström E, Lundgren P, Hård A-L, Lapillonne A, Sävman K, Nilsson AK, Andersson MX, Smith LEH, Hellström A (2017). Effects of a lipid emulsion containing fish oil on polyunsaturated fatty acid profiles, growth and morbidities in extremely premature infants: a randomized controlled trial. Clinical Nutrition ESPEN.

[bib137] Narayanan SP, Xu Z, Putluri N, Sreekumar A, Lemtalsi T, Caldwell RW, Caldwell RB (2014). Arginase 2 deficiency reduces hyperoxia-mediated retinal neurodegeneration through the regulation of polyamine metabolism. Cell Death & Disease.

[bib138] Nilsson AK, Löfqvist C, Najm S, Hellgren G, Sävman K, Andersson MX, Smith LEH, Hellström A (2019). Influence of human milk and parenteral lipid emulsions on serum fatty acid profiles in extremely preterm infants. JPEN. Journal of Parenteral and Enteral Nutrition.

[bib139] Nilsson AK, Andersson MX, Sjöbom U, Hellgren G, Lundgren P, Pivodic A, Smith LEH, Hellström A (2021). Sphingolipidomics of serum in extremely preterm infants: association between low sphingosine-1-phosphate levels and severe retinopathy of prematurity. Biochimica et Biophysica Acta. Molecular and Cell Biology of Lipids.

[bib140] Nilsson AK, Tebani A, Malmodin D, Pedersen A, Hellgren G, Löfqvist C, Hansen-Pupp I, Uhlén M, Hellström A (2022). Longitudinal serum metabolomics in extremely premature infants: relationships with gestational age, nutrition, and morbidities. Frontiers in Neuroscience.

[bib141] Ninchoji T, Love DT, Smith RO, Hedlund M, Vestweber D, Sessa WC, Claesson-Welsh L (2021). ENOS-induced vascular barrier disruption in retinopathy by c-src activation and tyrosine phosphorylation of VE-cadherin. eLife.

[bib142] Noguer MT, Martinez M (2010). Visual follow-up in peroxisomal-disorder patients treated with docosahexaenoic acid ethyl ester. Investigative Ophthalmology & Visual Science.

[bib143] Oei JL, Saugstad OD, Lui K, Wright IM, Smyth JP, Craven P, Wang YA, McMullan R, Coates E, Ward M, Mishra P, De Waal K, Travadi J, See KC, Cheah IGS, Lim CT, Choo YM, Kamar AA, Cheah FC, Masoud A, Tarnow-Mordi W (2017). Targeted oxygen in the resuscitation of preterm infants, a randomized clinical trial. Pediatrics.

[bib144] Oei JL, Vento M (2019). Is there a “right” amount of oxygen for preterm infant stabilization at birth?. Frontiers in Pediatrics.

[bib145] Öhnell HM, Andreasson S, Gränse L (2022). Dexamethasone eye drops for the treatment of retinopathy of prematurity. Ophthalmology. Retina.

[bib146] Oubaha M, Miloudi K, Dejda A, Guber V, Mawambo G, Germain M-A, Bourdel G, Popovic N, Rezende FA, Kaufman RJ, Mallette FA, Sapieha P (2016). Senescence-associated secretory phenotype contributes to pathological angiogenesis in retinopathy. Science Translational Medicine.

[bib147] Oziebło-Kupczyk M, Bakunowicz-Lazarczyk A, Dzienis K, Skrzydlewska E, Szczepański M, Waszkiewiczz E (2006). The estimation of selected parameters in antioxidant system in red blood cells in ROP screening of premature infants. Klinika Oczna.

[bib148] Palkovits S, Told R, Schmidl D, Boltz A, Napora KJ, Lasta M, Kaya S, Werkmeister RM, Popa-Cherecheanu A, Garhöfer G, Schmetterer L (2014). Regulation of retinal oxygen metabolism in humans during graded hypoxia. American Journal of Physiology. Heart and Circulatory Physiology.

[bib149] Paris LP, Johnson CH, Aguilar E, Usui Y, Cho K, Hoang LT, Feitelberg D, Benton HP, Westenskow PD, Kurihara T, Trombley J, Tsubota K, Ueda S, Wakabayashi Y, Patti GJ, Ivanisevic J, Siuzdak G, Friedlander M (2016). Global metabolomics reveals metabolic dysregulation in ischemic retinopathy. Metabolomics.

[bib150] Parra-Llorca A, Gormaz M, Alcántara C, Cernada M, Nuñez-Ramiro A, Vento M, Collado MC (2018). Preterm gut microbiome depending on feeding type: significance of donor human milk. Frontiers in Microbiology.

[bib151] Parrozzani R, Nacci EB, Bini S, Marchione G, Salvadori S, Nardo D, Midena E (2021). Severe retinopathy of prematurity is associated with early post-natal low platelet count. Scientific Reports.

[bib152] Pawlik D, Lauterbach R, Turyk E (2011). Fish-oil fat emulsion supplementation may reduce the risk of severe retinopathy in VLBW infants. Pediatrics.

[bib153] Pawlik D, Lauterbach R, Walczak M, Hurkała J, Sherman MP (2014). Fish-oil fat emulsion supplementation reduces the risk of retinopathy in very low birth weight infants: a prospective, randomized study. JPEN. Journal of Parenteral and Enteral Nutrition.

[bib154] Perkel JM (2021). Single-cell proteomics takes centre stage. Nature.

[bib155] Pesce NA, Canovai A, Plastino F, Lardner E, Kvanta A, Cammalleri M, André H, Dal Monte M (2021). An imbalance in autophagy contributes to retinal damage in a rat model of oxygen-induced retinopathy. Journal of Cellular and Molecular Medicine.

[bib156] Petit L, Ma S, Cipi J, Cheng SY, Zieger M, Hay N, Punzo C (2018). Aerobic glycolysis is essential for normal rod function and controls secondary cone death in retinitis pigmentosa. Cell Reports.

[bib157] Pierce EA, Foley ED, Smith LE (1996). Regulation of vascular endothelial growth factor by oxygen in a model of retinopathy of prematurity. Archives of Ophthalmology.

[bib158] Piñeiro-Ramos JD, Parra-Llorca A, Ten-Doménech I, Gormaz M, Ramón-Beltrán A, Cernada M, Quintás G, Collado MC, Kuligowski J, Vento M (2021). Effect of donor human milk on host-gut microbiota and metabolic interactions in preterm infants. Clinical Nutrition.

[bib159] Pivodic A, E H Smith L, Hård AL, Löfqvist C, Almeida AC, Al-Hawasi A, Larsson E, Lundgren P, Sunnqvist B, Tornqvist K, Wallin A, Holmstrom G, Gränse L (2022). Validation of DIGIROP models and decision support tool for prediction of treatment for retinopathy of prematurity on a contemporary swedish cohort. The British Journal of Ophthalmology.

[bib160] Rajala RVS, Rajala A, Kooker C, Wang Y, Anderson RE (2016). The warburg effect mediator pyruvate kinase M2 expression and regulation in the retina. Scientific Reports.

[bib161] Ramel SE, Long JD, Gray H, Durrwachter-Erno K, Demerath EW, Rao R (2013). Neonatal hyperglycemia and diminished long-term growth in very low birth weight preterm infants. Journal of Perinatology.

[bib162] Ramel S, Rao R (2020). Hyperglycemia in extremely preterm infants. NeoReviews.

[bib163] Rath PP, Jenkins S, Michaelides M, Smith A, Sweeney MG, Davis MB, Fitzke FW, Bird AC (2008). Characterisation of the macular dystrophy in patients with the A3243G mitochondrial DNA point mutation with fundus autofluorescence. The British Journal of Ophthalmology.

[bib164] Reading HW, Graymore CN (1965). Biochemistry of the Retina.

[bib165] Reidel B, Thompson JW, Farsiu S, Moseley MA, Skiba NP, Arshavsky VY (2011). Proteomic profiling of a layered tissue reveals unique glycolytic specializations of photoreceptor cells. Molecular & Cellular Proteomics.

[bib166] Rhee SY, Jung ES, Park HM, Jeong SJ, Kim K, Chon S, Yu SY, Woo JT, Lee CH (2018). Plasma glutamine and glutamic acid are potential biomarkers for predicting diabetic retinopathy. Metabolomics.

[bib167] Rinninella E, Mele MC, Merendino N, Cintoni M, Anselmi G, Caporossi A, Gasbarrini A, Minnella AM (2018). Retina axis. Nutrients.

[bib168] Riva CE, Grunwald JE, Sinclair SH (1983). Laser doppler velocimetry study of the effect of pure oxygen breathing on retinal blood flow. Investigative Ophthalmology & Visual Science.

[bib169] Roth AM (1977). Retinal vascular development in premature infants. American Journal of Ophthalmology.

[bib170] Rountree A, Karkamkar A, Khalil G, Folch A, Cook DL, Sweet IR (2016). BaroFuse, a novel pressure-driven, adjustable-throughput perfusion system for tissue maintenance and assessment. Heliyon.

[bib171] Şahinoğlu Keşkek N, Gülcan H, Yılmaz G, Akkoyun İ (2020). Impact of platelet count in retinopathy of prematurity. Turkish Journal of Ophthalmology.

[bib172] Salis ER, Reith DM, Wheeler BJ, Broadbent RS, Medlicott NJ (2017). Hyperglycaemic preterm neonates exhibit insulin resistance and low insulin production. BMJ Paediatrics Open.

[bib173] Sánchez-Infantes D, Gallego-Escuredo JM, Díaz M, Aragonés G, Sebastiani G, López-Bermejo A, de Zegher F, Domingo P, Villarroya F, Ibáñez L (2015). Circulating FGF19 and FGF21 surge in early infancy from infra- to supra-adult concentrations. International Journal of Obesity.

[bib174] SanGiovanni JP, Parra-Cabrera S, Colditz GA, Berkey CS, Dwyer JT (2000). Meta-Analysis of dietary essential fatty acids and long-chain polyunsaturated fatty acids as they relate to visual resolution acuity in healthy preterm infants. Pediatrics.

[bib175] Sapieha P, Sirinyan M, Hamel D, Zaniolo K, Joyal J-S, Cho J-H, Honoré J-C, Kermorvant-Duchemin E, Varma DR, Tremblay S, Leduc M, Rihakova L, Hardy P, Klein WH, Mu X, Mamer O, Lachapelle P, Di Polo A, Beauséjour C, Andelfinger G, Mitchell G, Sennlaub F, Chemtob S (2008). The succinate receptor GPR91 in neurons has a major role in retinal angiogenesis. Nature Medicine.

[bib176] Sato T, Wada K, Arahori H, Kuno N, Imoto K, Iwahashi-Shima C, Kusaka S (2012). Serum concentrations of bevacizumab (avastin) and vascular endothelial growth factor in infants with retinopathy of prematurity. American Journal of Ophthalmology.

[bib177] Scerri TS, Quaglieri A, Cai C, Zernant J, Matsunami N, Baird L, Scheppke L, Bonelli R, Yannuzzi LA, Friedlander M, Egan CA, Fruttiger M, Leppert M, Allikmets R, Bahlo M, MacTel Project Consortium (2017). Genome-wide analyses identify common variants associated with macular telangiectasia type 2. Nature Genetics.

[bib178] Schoors S, De Bock K, Cantelmo AR, Georgiadou M, Ghesquière B, Cauwenberghs S, Kuchnio A, Wong BW, Quaegebeur A, Goveia J, Bifari F, Wang X, Blanco R, Tembuyser B, Cornelissen I, Bouché A, Vinckier S, Diaz-Moralli S, Gerhardt H, Telang S, Cascante M, Chesney J, Dewerchin M, Carmeliet P (2014). Partial and transient reduction of glycolysis by PFKFB3 blockade reduces pathological angiogenesis. Cell Metabolism.

[bib179] Schoors S, Bruning U, Missiaen R, Queiroz KC, Borgers G, Elia I, Zecchin A, Cantelmo AR, Christen S, Goveia J, Heggermont W, Goddé L, Vinckier S, Van Veldhoven PP, Eelen G, Schoonjans L, Gerhardt H, Dewerchin M, Baes M, De Bock K, Ghesquière B, Lunt SY, Fendt S-M, Carmeliet P (2015). Fatty acid carbon is essential for dntp synthesis in endothelial cells. Nature.

[bib180] Seliniotaki AK, Haidich AB, Moutzouri S, Lithoxopoulou M, Ziakas N, Lundgren P, Hellström A, Mataftsi A (2022). Association of platelet deficiency with severe retinopathy of prematurity: a review. Acta Paediatrica.

[bib181] Sennlaub F, Courtois Y, Goureau O (2002). Inducible nitric oxide synthase mediates retinal apoptosis in ischemic proliferative retinopathy. The Journal of Neuroscience.

[bib182] Seydel C (2021). Single-Cell metabolomics hits its stride. Nature Methods.

[bib183] Shao Z, Dorfman AL, Seshadri S, Djavari M, Kermorvant-Duchemin E, Sennlaub F, Blais M, Polosa A, Varma DR, Joyal J-S, Lachapelle P, Hardy P, Sitaras N, Picard E, Mancini J, Sapieha P, Chemtob S (2011). Choroidal involution is a key component of oxygen-induced retinopathy. Investigative Ophthalmology & Visual Science.

[bib184] Shih S-C, Ju M, Liu N, Smith LEH (2003). Selective stimulation of VEGFR-1 prevents oxygen-induced retinal vascular degeneration in retinopathy of prematurity. The Journal of Clinical Investigation.

[bib185] Shukla A, Sonnie C, Worley S, Sharma A, Howard D, Moore J, Rodriguez RJ, Hoppe G, Sears JE (2019). Comparison of biphasic vs static oxygen saturation targets among infants with retinopathy of prematurity. JAMA Ophthalmology.

[bib186] Sinclair R, Schindler T, Lui K, Bolisetty S (2018). Hypertriglyceridaemia in extremely preterm infants receiving parenteral lipid emulsions. BMC Pediatrics.

[bib187] Singh C, Hoppe G, Tran V, McCollum L, Bolok Y, Song W, Sharma A, Brunengraber H, Sears JE (2019). Serine and 1-carbon metabolism are required for HIF-mediated protection against retinopathy of prematurity. JCI Insight.

[bib188] Skondra D, Rodriguez SH, Sharma A, Gilbert J, Andrews B, Claud EC (2020). The early gut microbiome could protect against severe retinopathy of prematurity. Journal of AAPOS.

[bib189] Smith LE, Shen W, Perruzzi C, Soker S, Kinose F, Xu X, Robinson G, Driver S, Bischoff J, Zhang B, Schaeffer JM, Senger DR (1999). Regulation of vascular endothelial growth factor-dependent retinal neovascularization by insulin-like growth factor-1 receptor. Nature Medicine.

[bib190] Smith SL, Rouse CA (2017). Docosahexaenoic acid and the preterm infant. Maternal Health, Neonatology and Perinatology.

[bib191] Solaini G, Baracca A, Lenaz G, Sgarbi G (2010). Hypoxia and mitochondrial oxidative metabolism. Biochimica et Biophysica Acta.

[bib192] Spierer A, Rabinowitz R, Pri-Chen S, Rosner M (2005). An increase in superoxide dismutase ameliorates oxygen-induced retinopathy in transgenic mice. Eye.

[bib193] Sprott D, Poitz DM, Korovina I, Ziogas A, Phieler J, Chatzigeorgiou A, Mitroulis I, Deussen A, Chavakis T, Klotzsche-von Ameln A (2019). Endothelial-specific deficiency of ATG5 (autophagy protein 5) attenuates ischemia-related angiogenesis. Arteriosclerosis, Thrombosis, and Vascular Biology.

[bib194] Stahl A, Krohne TU, Eter N, Oberacher-Velten I, Guthoff R, Meltendorf S, Ehrt O, Aisenbrey S, Roider J, Gerding H, Jandeck C, Smith LEH, Walz JM, Comparing Alternative Ranibizumab Dosages for Safety and Efficacy in Retinopathy of Prematurity Study Group (2018). Comparing alternative ranibizumab dosages for safety and efficacy in retinopathy of prematurity: A randomized clinical trial. JAMA Pediatrics.

[bib195] Stahl A, Sukgen EA, Wu W-C, Lepore D, Nakanishi H, Mazela J, Moshfeghi DM, Vitti R, Athanikar A, Chu K, Iveli P, Zhao F, Schmelter T, Leal S, Köfüncü E, Azuma N, FIREFLEYE Study Group (2022). Effect of intravitreal aflibercept vs laser photocoagulation on treatment success of retinopathy of prematurity: the FIREFLEYE randomized clinical trial. JAMA.

[bib196] Sun Y, Ju M, Lin Z, Fredrick TW, Evans LP, Tian KT, Saba NJ, Morss PC, Pu WT, Chen J, Stahl A, Joyal J-S, Smith LEH (2015). Socs3 in retinal neurons and glial cells suppresses VEGF signaling to prevent pathological neovascular growth. Science Signaling.

[bib197] Sun Y, Lin Z, Liu C-H, Gong Y, Liegl R, Fredrick TW, Meng SS, Burnim SB, Wang Z, Akula JD, Pu WT, Chen J, Smith LEH (2017). Inflammatory signals from photoreceptor modulate pathological retinal angiogenesis via c-fos. The Journal of Experimental Medicine.

[bib198] Talukdar S, Zhou Y, Li D, Rossulek M, Dong J, Somayaji V, Weng Y, Clark R, Lanba A, Owen BM, Brenner MB, Trimmer JK, Gropp KE, Chabot JR, Erion DM, Rolph TP, Goodwin B, Calle RA (2016). A long-acting FGF21 molecule, PF-05231023, decreases body weight and improves lipid profile in non-human primates and type 2 diabetic subjects. Cell Metabolism.

[bib199] Tao K (2022). Postnatal administration of systemic steroids increases severity of retinopathy in premature infants. Pediatrics and Neonatology.

[bib200] Tin W, Milligan DW, Pennefather P, Hey E (2001). Pulse oximetry, severe retinopathy, and outcome at one year in babies of less than 28 weeks gestation. Archives of Disease in Childhood. Fetal and Neonatal Edition.

[bib201] Tomita Y, Cagnone G, Fu Z, Cakir B, Kotoda Y, Asakage M, Wakabayashi Y, Hellström A, Joyal JS, Talukdar S, Smith LEH, Usui Y (2021a). Vitreous metabolomics profiling of proliferative diabetic retinopathy. Diabetologia.

[bib202] Tomita Y, Usui-Ouchi A, Nilsson AK, Yang J, Ko M, Hellström A, Fu Z (2021b). Metabolism in retinopathy of prematurity. Life.

[bib203] Tsantilas KA, Cleghorn WM, Bisbach CM, Whitson JA, Hass DT, Robbings BM, Sadilek M, Linton JD, Rountree AM, Valencia AP, Sweetwyne MT, Campbell MD, Zhang H, Jankowski CSR, Sweet IR, Marcinek DJ, Rabinovitch PS, Hurley JB (2021). An analysis of metabolic changes in the retina and retinal pigment epithelium of aging mice. Investigative Ophthalmology & Visual Science.

[bib204] Ueda K, Nakahara T, Hoshino M, Mori A, Sakamoto K, Ishii K (2010). Retinal blood vessels are damaged in a rat model of NMDA-induced retinal degeneration. Neuroscience Letters.

[bib205] Vandekeere S, Dubois C, Kalucka J, Sullivan MR, García-Caballero M, Goveia J, Chen R, Diehl FF, Bar-Lev L, Souffreau J, Pircher A, Kumar S, Vinckier S, Hirabayashi Y, Furuya S, Schoonjans L, Eelen G, Ghesquière B, Keshet E, Li X, Vander Heiden MG, Dewerchin M, Carmeliet P (2018). Serine synthesis via Phgdh is essential for heme production in endothelial cells. Cell Metabolism.

[bib206] Vanhaesebrouck S, Vanhole C, de Zegher F, Allegaert K (2008). Influence of duration of parenteral nutrition on retinopathy of prematurity. Archives of Disease in Childhood. Fetal and Neonatal Edition.

[bib207] Vanhaesebrouck S, Daniëls H, Moons L, Vanhole C, Carmeliet P, De Zegher F (2009). Oxygen-induced retinopathy in mice: amplification by neonatal IGF-I deficit and attenuation by IGF-I administration. Pediatric Research.

[bib208] Vento M, Cubells E, Escobar JJ, Escrig R, Aguar M, Brugada M, Cernada M, Saénz P, Izquierdo I (2013). Oxygen saturation after birth in preterm infants treated with continuous positive airway pressure and air: assessment of gender differences and comparison with a published nomogram. Archives of Disease in Childhood. Fetal and Neonatal Edition.

[bib209] Villarejo-Zori B, Jiménez-Loygorri JI, Zapata-Muñoz J, Bell K, Boya P (2021). New insights into the role of autophagy in retinal and eye diseases. Molecular Aspects of Medicine.

[bib210] Wallace DK, Dean TW, Hartnett ME, Kong L, Smith LE, Hubbard GB, McGregor ML, Jordan CO, Mantagos IS, Bell EF, Kraker RT, Pediatric Eye Disease Investigator Group (2018). A dosing study of bevacizumab for retinopathy of prematurity: late recurrences and additional treatments. Ophthalmology.

[bib211] Wang L, Törnquist P, Bill A (1997). Glucose metabolism in pig outer retina in light and darkness. Acta Physiologica Scandinavica.

[bib212] Wang J, Xu X, Elliott MH, Zhu M, Le YZ (2010). Müller cell-derived VEGF is essential for diabetes-induced retinal inflammation and vascular leakage. Diabetes.

[bib213] Wangsa-Wirawan ND, Linsenmeier RA (2003). Retinal oxygen: fundamental and clinical aspects. Archives of Ophthalmology.

[bib214] Webbe J, Uthaya S, Modi N (2022). Nutrition for the micro preemie: beyond milk. Seminars in Fetal & Neonatal Medicine.

[bib215] Wei X, Schneider JG, Shenouda SM, Lee A, Towler DA, Chakravarthy MV, Vita JA, Semenkovich CF (2011). De novo lipogenesis maintains vascular homeostasis through endothelial nitric-oxide synthase (eNOS) palmitoylation. The Journal of Biological Chemistry.

[bib216] Westaway JAF, Huerlimann R, Kandasamy Y, Miller CM, Norton R, Staunton KM, Watson D, Rudd D (2022). The bacterial gut microbiome of probiotic-treated very-preterm infants: changes from admission to discharge. Pediatric Research.

[bib217] Wheaton WW, Chandel NS (2011). Hypoxia. 2. hypoxia regulates cellular metabolism. American Journal of Physiology. Cell Physiology.

[bib218] Whitfield JM, Hendrikson H (2006). Prevention of protein deprivation in the extremely low birth weight infant: a nutritional emergency. Proceedings.

[bib219] Wu W-C, Lien R, Liao P-J, Wang N-K, Chen Y-P, Chao A-N, Chen K-J, Chen T-L, Hwang Y-S, Lai C-C (2015). Serum levels of vascular endothelial growth factor and related factors after intravitreous bevacizumab injection for retinopathy of prematurity. JAMA Ophthalmology.

[bib220] Xu Y, An X, Guo X, Habtetsion TG, Wang Y, Xu X, Kandala S, Li Q, Li H, Zhang C, Caldwell RB, Fulton DJ, Su Y, Hoda MN, Zhou G, Wu C, Huo Y (2014). Endothelial PFKFB3 plays a critical role in angiogenesis. Arteriosclerosis, Thrombosis, and Vascular Biology.

[bib221] Yagasaki R, Nakahara T, Ushikubo H, Mori A, Sakamoto K, Ishii K (2014). Anti-Angiogenic effects of mammalian target of rapamycin inhibitors in a mouse model of oxygen-induced retinopathy. Biological & Pharmaceutical Bulletin.

[bib222] Yang Y, Wu Z, Li S, Yang M, Xiao X, Lian C, Wen W, He H, Zeng J, Wang J, Zhang G (2020). Targeted blood metabolomic study on retinopathy of prematurity. Investigative Ophthalmology & Visual Science.

[bib223] Yang Y, Yang Q, Luo S, Zhang Y, Lian C, He H, Zeng J, Zhang G (2022). Comparative analysis reveals novel changes in plasma metabolites and metabolomic networks of infants with retinopathy of prematurity. Investigative Ophthalmology & Visual Science.

[bib224] Yossuck P, Yan Y, Tadesse M, Higgins RD (2000). Dexamethasone and critical effect of timing on retinopathy. Investigative Ophthalmology & Visual Science.

[bib225] Yossuck P, Yan Y, Tadesse M, Higgins RD (2001). Dexamethasone alters TNF-alpha expression in retinopathy. Molecular Genetics and Metabolism.

[bib226] Yu-Wai-Man P, Newman NJ (2017). Inherited eye-related disorders due to mitochondrial dysfunction. Human Molecular Genetics.

[bib227] Zhang Q, Zhang J, Guan Y, Zhang S, Zhu C, Xu GT, Wang L (2009). Suppression of retinal neovascularization by the iNOS inhibitor aminoguanidine in mice of oxygen-induced retinopathy. Graefe’s Archive for Clinical and Experimental Ophthalmology = Albrecht von Graefes Archiv Fur Klinische Und Experimentelle Ophthalmologie.

[bib228] Zhang JY, Xie B, Barba H, Nadeem U, Movahedan A, Deng N, Spedale M, D’Souza M, Luo W, Leone V, Chang EB, Theriault B, Sulakhe D, Skondra D (2022). Absence of gut microbiota is associated with RPE/choroid transcriptomic changes related to age-related macular degeneration pathobiology and decreased choroidal neovascularization. International Journal of Molecular Sciences.

[bib229] Zhao C, Yasumura D, Li X, Matthes M, Lloyd M, Nielsen G, Ahern K, Snyder M, Bok D, Dunaief JL, LaVail MM, Vollrath D (2011). Mtor-Mediated dedifferentiation of the retinal pigment epithelium initiates photoreceptor degeneration in mice. The Journal of Clinical Investigation.

[bib230] Zheng L, Gong B, Hatala DA, Kern TS (2007). Retinal ischemia and reperfusion causes capillary degeneration: similarities to diabetes. Investigative Ophthalmology & Visual Science.

